# Identification of key regions and residues controlling Aβ folding and assembly

**DOI:** 10.1038/s41598-017-10845-6

**Published:** 2017-10-03

**Authors:** Eric Y. Hayden, Kimberly K. Hoi, Jasmine Lopez, Mohammed Inayathullah, Margaret M. Condron, David B. Teplow

**Affiliations:** 1 Department of Neurology, David Geffen School of Medicine at UCLA, Los Angeles, CA 90095 USA; 2 Molecular Biology Institute and Brain Research Institute; UCLA, Los Angeles, CA 90095 USA; 3 Biomaterials and Advanced Drug Delivery Laboratory, School of Medicine, Stanford University, Palo Alto, CA 94304 USA; 4 Present Address: Department of Pediatrics and Department of Neurology, UCSF, San Francisco, CA 94158 USA

## Abstract

Amyloid β-protein (Aβ) assembly is hypothesized to be a seminal neuropathologic event in Alzheimer’s disease (AD). We used an unbiased D-amino acid substitution strategy to determine structure-assembly relationships of 76 different Aβ40 and Aβ42 peptides. We determined the effects of the substitutions on peptide oligomerization, secondary structure dynamics, fibril assembly dynamics, and fibril morphology. Our experiments revealed that the assembly of Aβ42 was more sensitive to chiral substitutions than was Aβ40 assembly. Substitutions at identical positions in the two peptides often, but not always, produced the same effects on assembly. Sites causing substantial effects in both Aβ40 and Aβ42 include His14, Gln15, Ala30, Ile31, Met35, and Val36. Sites whose effects were unique to Aβ40 include Lys16, Leu17, and Asn 27, whereas sites unique to Aβ42 include Phe20 and Ala21. These sites may be appropriate targets for therapeutic agents that inhibit or potentiate, respectively, these effects.

## Introduction

Alzheimer’s disease (AD) is the most common cause of late-life dementia^1^ and recently has been identified as the 6th leading cause of death in the U.S.^2^. Thus, there is a compelling need for the development of approaches to prevent, ameliorate, or cure this tragic disorder. A predominant working hypothesis of disease causation posits that oligomeric forms of the amyloid β-protein (Aβ) are key neurotoxic agents^3^. If so, then therapeutic drug development requires appropriate targeting of these assemblies. A variety of targeting strategies have been executed, including those directed against the enzymes responsible for Aβ production (β-secretase and γ-secretase)^4^ or immunogenic sites on monomeric, oligomeric, and fibrillar forms of Aβ^5^. Unfortunately, none have resulted in an FDA-approved therapeutic agent^6^.

Aβ exists in humans predominantly in two forms, Aβ40 and Aβ42, that are 40 or 42 amino acid residues in length, respectively^4^. Aβ42 appears to be the most disease-relevant peptide^7–9^. Mutations in the gene encoding the amyloid precursor protein (APP), from which Aβ42 is produced, lead to single amino acid substitutions linked to familial forms of AD, cerebral amyloid angiopathy (CAA), or AD with CAA. Most mutations result in single amino acid substitutions^10–22^. One results in the deletion of Glu22^10^. *In vitro* studies of the conformational dynamics and assembly of Aβ peptides containing these substitutions show that they facilitate folding, oligomerization, or fibril formation by the initially disordered Aβ monomer (for reviews, see refs^23–25^). However, familial AD (with or without CAA) is estimated to account for <1% of all AD cases^26^. This means that in the majority of AD cases, Aβ-mediated neurotoxicity and plaque formation are caused by wild type Aβ.

We sought here to determine which amino acids in wild type Aβ40 and Aβ42 have the greatest effects on peptide folding and assembly. Prior approaches for answering this question often have relied on amino acid substitution strategies, which have been informative and useful. However, by definition, the substituted amino acids differ from the wild type amino acids in polarity, charge, hydrophobicity, or size of the amino acid side-chains. These differences *per se* may be responsible for any changes, or lack of changes, in peptide folding and assembly, as opposed to the differences indicating how the wild type amino is involved these processes. To avoid these interpretive difficulties, we employed a scanning D-amino acid substitution strategy. We did so because chiral substitution only affects the orientation of the side-chain relative to the peptide backbone^27–30^. These substitutions thus reveal amino acids whose side-chains are involved in inter-atomic interactions that are exquisitely sensitive to structural perturbation and thus may be of special importance in controlling Aβ assembly and toxicity. In much the same way that study of transition states in chemical and enzymatic reactions are critical for establishing a mechanistic understanding of such reactions, chiral inversions may enable the study of peptide folding trajectories rarely traversed by the wild type peptide but that are critical for the production of conformers associated with pathologic Aβ assembly^31,32^ (see Raskatov and Teplow^30^ for a theoretical treatment of the scanning D-amino acid strategy). Our experimental design comprised initial studies of the effects of scanning di-D amino acid substitution on peptide oligomerization and fibril formation, followed by determination of the effects of single D-amino acid substitutions chosen based on the data obtained in the initial studies. This allowed us to determine the effects of specific amino acids on peptide oligomerization, secondary structure, fibril assembly, and fibril morphology and if any differences in effects were observed between Aβ40 and Aβ42.

## Materials and Methods

### Peptide synthesis and preparation

Aβ40, Aβ42, di-D-amino acid substituted peptides, and single D-amino acid substituted peptides were synthesized, purified, and characterized in the Biopolymer Lab at UCLA^33^. Briefly, peptide synthesis was performed on an automated peptide synthesizer (Model 433 A, Applied Biosystems, Foster City, CA) using 9-fluorenylmethoxycarbonyl-based methods at a 0.25 mmol scale. Peptides were purified using reverse phase high-performance liquid chromatography (RP-HPLC). The purity of the peptides were >95%. Quantitative amino acid analysis and mass spectrometry yielded the expected compositions and molecular weights, respectively, for each peptide. Purified peptides were stored as lyophilizates at −20 °C.

Peptide lyophilizates were solvated initially in 10% (v/v) 60 mM NaOH, 45% (v/v) H_2_O (prepared using a Synthesis A10 water purification system (Millipore, Bedford, MA)), and 45% (v/v) 22.2 mM sodium phosphate, pH 7.4, on ice. The solutions were sonicated for 1 min in an ultrasonic water bath (Model 1510, Branson Ultrasonics Corp., Danbury, CT) and then they were filtered using a Microcon centrifugal filter (30 kDa molecular mass cut-off, Millipore, Bedford, MA) at 14,000 × *g* for 10 min at room temperature (RT ≈22 °C) The concentrations of the resulting filtrates were determined by UV absorbance using an extinction coefficient ε_276_ = 1276 L mol^−1^ cm^−1^ (tyrosine absorption maximum is 276 nm^34^). The peptide concentration then was adjusted to 40 μM using 10 mM sodium phosphate, pH 7.4.

### Cross-linking and SDS-PAGE analysis

Aβ peptides were covalently cross-linked using the technique of photo-induced cross-linking of unmodified proteins (PICUP), as described^35^. Briefly, 1 μL of 40 mM ammonium persulfate (APS) and 1 μL of 2 mM Tris(2,2′- bipyridyl)dichlororuthenium(II)) were added to 18 μL of 40 μM Aβ. The mixture was irradiated for 1 s with visible light (Dolan-Jenner Industries Fiber-Lite Model 170-D, Boxborough, MA) and the reaction was quenched immediately with 1 μL of 1 M dithiothreitol in H_2_O. Tricine SDS Sample Buffer (2X) (Invitrogen, Carlsbad, CA) was then added to the solution. Five µL of each cross-linked Aβ was analyzed by SDS-PAGE using 10–20% Tricine gels (1.0 mm × 12 well) and subsequently silver stained following the Silver Xpress Silver Staining Protocol (Invitrogen). Gels were dried overnight using Novex DryEase Mini cellophane in Novex drying frames and then scanned with a Canon CanoScan 9950 F flatbed scanner at 300 dpi or greater. Three independent experiments were performed with each peptide. We determined the relative intensities of each band in each lane by performing densitometry using ImageJ 1.50d (http://imagej.nih.gov/ij) and then calculating the normalized intensity of each band ${N}_{i}={I}_{i}/{\sum }_{1}^{n}{I}_{i}$; where *I*_*i*_ is the intensity of band *i* and ${\sum }_{1}^{n}{I}_{i}$ is the sum of all band intensities. We then calculated the absolute values of the differences between the intensities of each oligomer band in the wild type peptides and the corresponding bands in the substituted peptides. These differences were summed to produce a “difference metric” for determination of the magnitudes of the differences between wild type and substituted peptides.

### Thioflavin T fluorescence

Aβ42 and its variants were initially dissolved in ≈1 mL of 4 °C hexaflouro-2-propanol (HFIP), sonicated 5 min to completely dissolve the Aβ, incubated for 30 min incubation at RT, and aliquoted into a 1.5 mL low retention microcentrifuge tube (FisherBrand). The HFIP was completely evaporated overnight in a chemical fume hood. The following day, the tubes were rotary evaporated for 1 h in a SpeedVac concentrator (Thermo Scientific, Savant SPD121P), which ensured complete removal of the HFIP. The peptides within the films then were prepared for use as described above for peptide lyophilizates.

Immediately after sample preparation, 100 μL aliquots of each of the peptides (40 μM Aβ40, 20 μM Aβ42) were mixed with 100 μL of 120 μM ThT in 10 mM sodium phosphate, pH 7.4, in wells of a Thermo Scientific™ Nunc™ Microwell™ 96-Well optical-bottom plate. The plate was sealed with SealPlate film (Excel Scientific) and then incubated at 37 °C with shaking at 160 rpm. ThT fluorescence then was measured using a BioTek Synergy HT plate reader (BioTek Instruments, Inc., Winooski, VT, USA) with excitation and emission filters set to 420 nm and 485 nm, respectively. Slit-widths for excitation and emission were 50 and 20 nm, respectively. Three independent experiments were performed for each peptide. The time at which half-maximal ThT intensity (t_1/2_) was observed was determined by calculating the difference between the final and initial fluorescence levels, visually locating that value on a smooth fit of the graph of the time-dependence of fluorescence intensity, and then identifying the corresponding time. Lag times were defined as the time at which exponential increases in fluorescence began to be observed. They were determined by identifying the x-intercept of a line fitted to the quasi-linear phase of the increase in fluorescence between the starting and plateau fluorescence levels. We define the change in fluorescence intensity per unit time (dFU/dt) as the slope of the line fitted to the quasi-linear phase of growth of each curve of fluorescence intensity versus time.

### Circular dichroism spectroscopy (CD)

Peptides were prepared as described above for the ThT experiments. Spectra were acquired periodically during incubation of the peptides at 37 °C, without agitation, in 0.1 cm path-length quartz cuvettes (Hellma, Forest Hills, NY). Spectra were acquired using a Jasco Model J-810 spectropolarimeter (Jasco Corporation, Japan) over a wavelength range of 195–260 nm. Parameters for measurements were: standard sensitivity, data pitch = 0.2 nm, scanning speed = 100 nm/min, and bandwidth = 1 nm. Ten spectra were taken per sample. Three independent experiments were performed with each peptide.

### Transmission electron microscopy (TEM)

Aliquots of 8 µL volume were removed periodically from assembly reactions being monitored by CD. Each aliquot was applied to 400 mesh carbon-coated formvar EM grids (Electron Microscopy Sciences, Hatfield, PA). Each grid was incubated for 2 min at RT. The liquid was then wicked off by gently bringing the tip of 55 mm diameter, #2 filter paper (Whatman) to the edge of the grid. Subsequently 8 μL of 1% (w/v) uranyl acetate in MilliQ water was applied to the grid and immediately wicked off. A JEOL 1200 EX transmission electron microscope was used to visualize the sample morphologies.

## Results

### Experimental strategy

**Figure 1:**
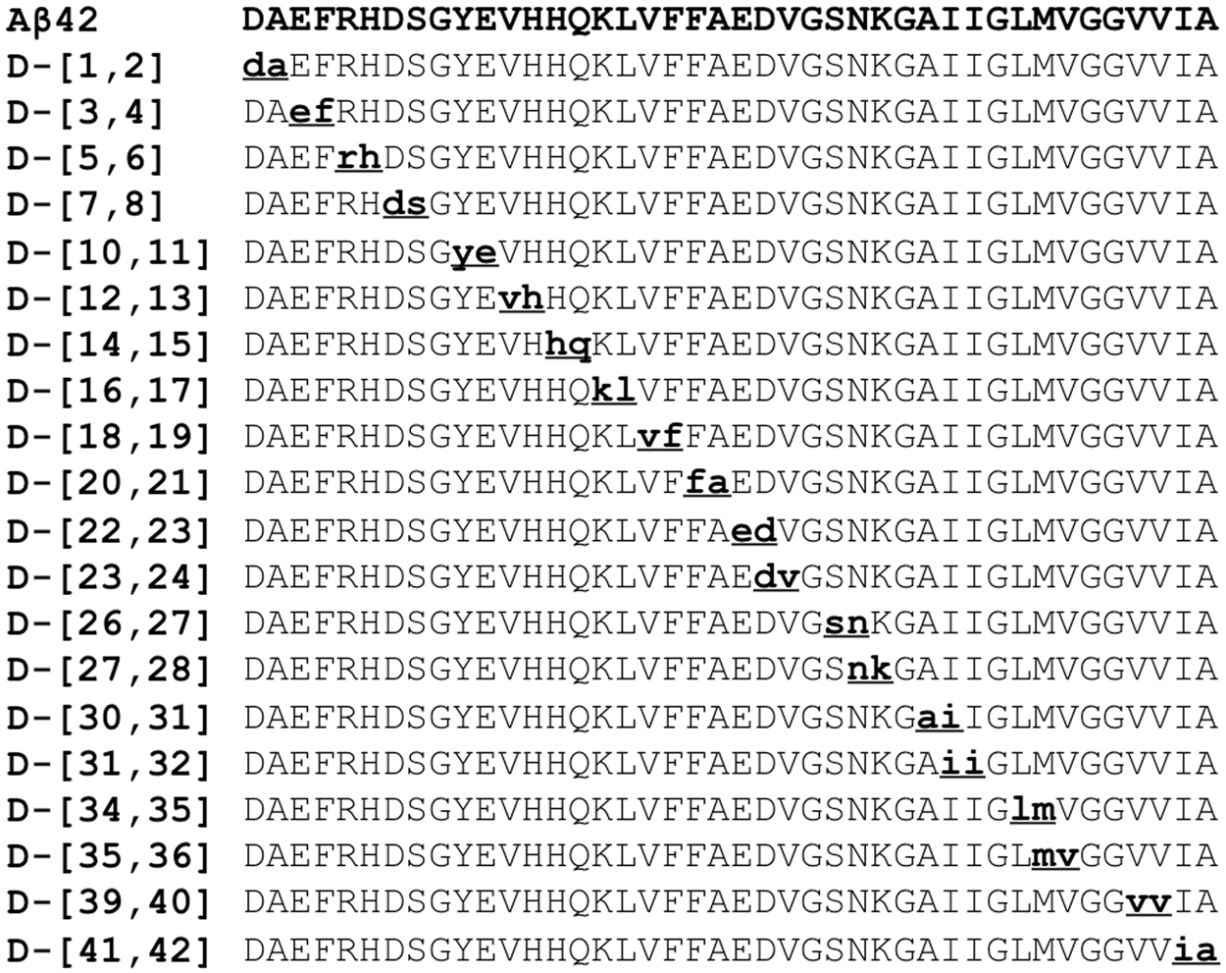
Location of di-D-amino acid substitutions. Substitutions are indicated by bolded, underlined, lower-case letters. The substitutions were identical for Aβ40 and Aβ42, except for the additional **ia** at the 41 and 42 positions in Aβ42.

We executed a scanning D-amino acid substitution strategy in two phases that involved the synthesis and study of a total of 76 different D-amino acid substituted Aβ peptides. Single D-amino acid replacements at each position along amphipathic model peptides have shown that a single replacement may not always be sufficient for a reliable determination of a structural effect^28^. Pairwise substitution of adjacent amino acids by their corresponding D-amino acids produces a more substantial structural change^27^. For these reasons, we first synthesized all possible di-D-amino acid-substituted Aβ40 and Aβ42 isomers (Fig. 1). Positions containing Gly residues were not included as members of any di-substituted sites because this amino acid is achiral. Each of these initial 39 peptides (19 Aβ40 peptides and 20 Aβ42 peptides) was monitored for effects of the substitutions on oligomerization and fibril formation. If effects were observed for a particular di-D-amino acid substitution, then two additional peptides were synthesized, each containing only one of the two amino acids comprising the original di-D-amino acid segment. For select mono-substituted peptides, in addition to monitoring oligomerization and fibril formation, CD was used to study time-dependent changes in secondary structure and TEM was used to determine assembly morphology.

### Oligomerization of di-D-amino acid substituted peptides

**Figure 2:**
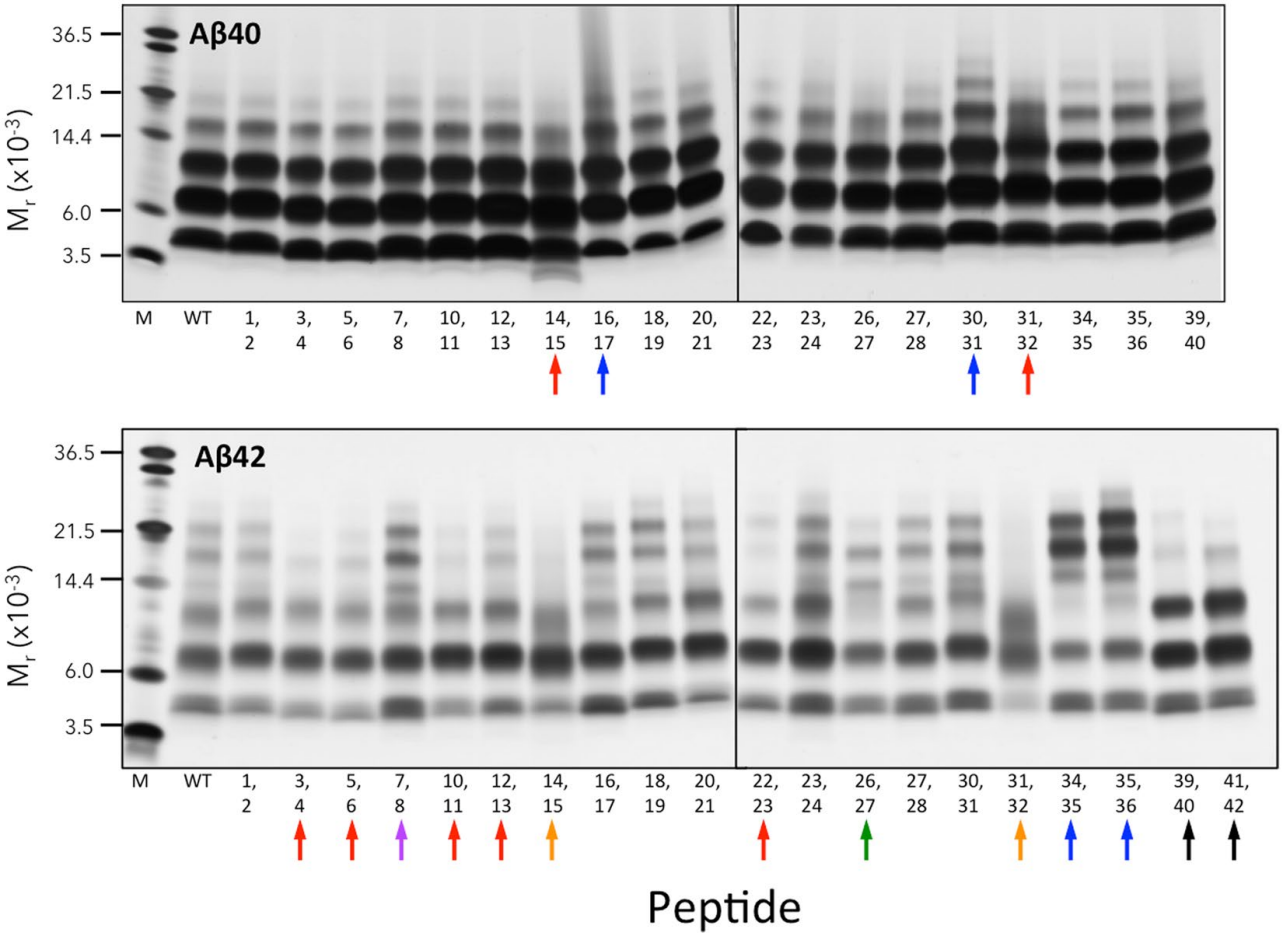
Oligomerization of di-D-amino acid substituted Aβ. Peptides were cross-linked using PICUP. SDS-PAGE and silver staining were then performed to reveal the effects of D-amino acid substitutions on Aβ40 and Aβ42 oligomerization. M_r_ indicates apparent molecular weight. Lanes “M” are molecular weight markers. WT indicates the wild type peptide. Positions of D-amino acid substitutions are indicated by numbers below each gel. Colored arrows represent different classes of oligomerization (see text). Gels are representative of results in each of three independent experiments.

Photo-induced cross-linking of unmodified proteins (PICUP) was used to stabilize oligomer states, allowing quantitative determination of the oligomer size frequency distribution using SDS-PAGE, silver staining, and densitometry. Aβ40 produced an oligomer distribution comprising predominately dimers and trimers, with progressively fewer tetramers and pentamers (Fig. 2; Aβ40, lane “WT”). Several di-D-substituted Aβ40 peptides displayed altered oligomer distributions. For example, D-[H14,Q15] displayed a prominent band with a mobility greater than that of monomer, as well as a more diffuse and fainter tetramer band (Fig. 2, red arrow). [For ease of reference, we refer to particular peptides by specifying only the position(s) of D-amino acids. Thus D-[H14,Q15] refers to a full-length Aβ peptide containing D-His14 and D-Q15.] The oligomer distribution of D-[K16,L17] was shifted to higher oligomer orders, as evidenced by a more prominent pentamer band, a diffuse hexamer band, and poorly resolved higher order bands. D-[A30,I31], like D-[K16,L17], had an oligomer distribution shifted to higher orders, but in this case, bands were distinct, not smeared. D-[A31,I32] showed substantial smearing between trimer and tetramer.

Substituted Aβ42 peptides produced six different classes of oligomer distributions (Fig. 2, Aβ42 panel, colored arrows), compared with two in the Aβ40 case. The largest class (red) was characterized by decreased amounts of pentamers and hexamers. The orange class (D-[H14,Q15] and D-[I31,I32]), in addition to displaying exceptionally faint pentamer and hexamer bands, produced a diffuse band with an M_r_ most consistent with trimer, but with substantial areas of this band migrating at significantly lower M_r_. The dimer band also was diffuse. D-[D7, S8] (purple arrow) was unique in that it showed increased intensity of tetramer, pentamer, and hexamer. The D-[I31,I32] peptide was notable because it displayed the lowest amount of monomer relative to the other bands in the lane. D-[S26,N27] was unique in that decreases in hexamer and trimer band intensities were observed in concert with increased tetramer intensity (green arrow). D-[L34,M35] and D-[M35,V36] peptides displayed exceptionally intense pentamer and hexamer bands, but less intense trimer bands (blue arrows). D-[V39,V40] and D-[I41,A42] showed exceptionally intense dimer and trimer bands, but tetramers were difficult to detect (black arrows).

### Thioflavin T (ThT) fluorescence of di-D-amino acid substituted Aβ

**Figure 3:**
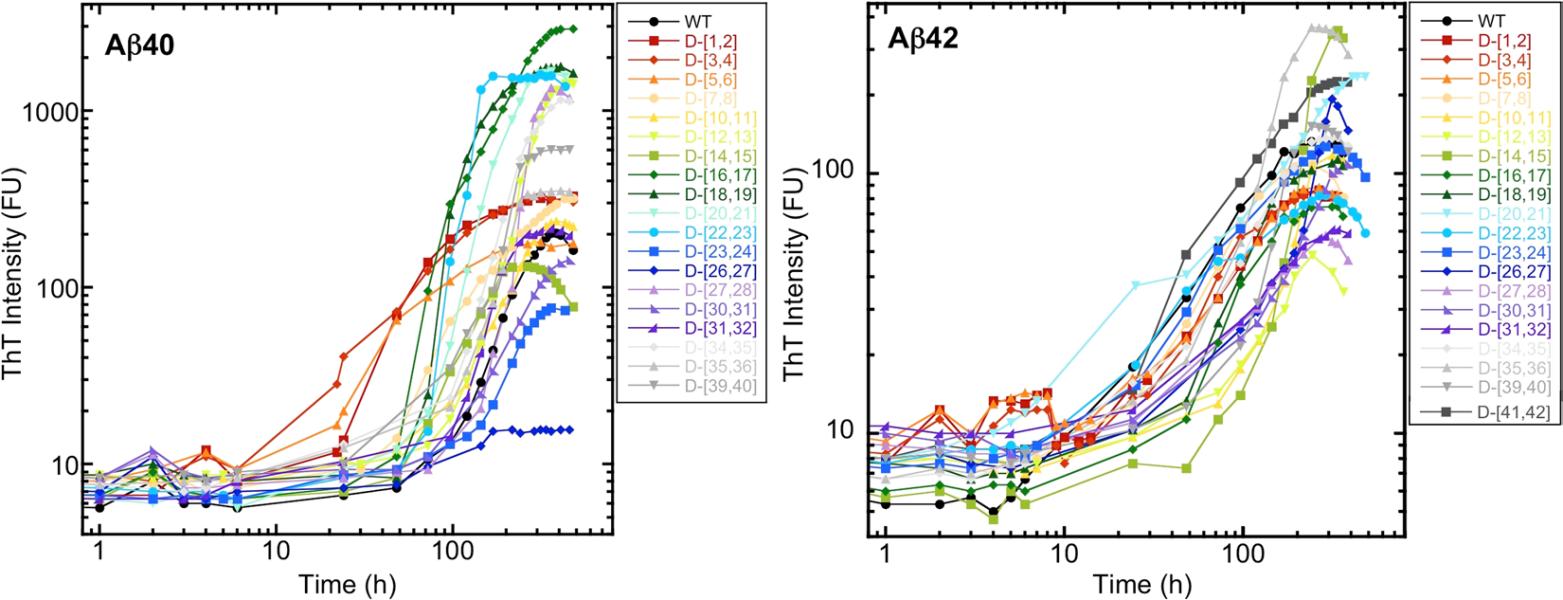
Fibril formation kinetics of di-D-amino acid substituted Aβ. Peptides (40 μM Aβ40, 20 μM Aβ42) were mixed with Thioflavin T in 10 mM sodium phosphate, pH 7.4, and incubated with shaking at 37 °C. (**A**) Aβ40 with di-D-amino acid substitutions. (**B**) Aβ42 with di-D-amino acid substitutions. The peptides examined are shown in the boxes to the right of each sub-figure. Note that log-log plots are shown. (Fig. S1 shows semi-log plots of the same data.)

To determine the effects of di-D-amino acid substitutions on the time dependence of β-sheet formation, a proxy for Aβ fibril formation^36–38^, we monitored Thioflavin T (ThT) fluorescence (Fig. 3). The average maximum intensity (FU_max_) of Aβ40 was 200 fluorescence units (FU) (Table 1). Aβ40 variants D-[V12,H13], D-[K16,L17], D-[V18,F19], D-[F20,A21], D-[E22,D23], D-[N27,K28], D-[L34,M35], and D-[V39,V40] displayed substantially greater intensities. To avoid over interpretation of the data, we define “substantial” as <1/2× or >2× the wild type intensity. D-[Asp1,Ala2], D-[Glu3, Phe4], and D-[Asp7,Ser8] had very similar maximum intensities, all of which were ≈1.5× that of wild type Aβ40. The intensities of D-[Asp23,Val24] and D-[Ser26, Asn27] were substantially lower ~1/3 and <1/10, respectively, than that of wild type Aβ40. The time at which half maximal fluorescence intensity occurred (t_1/2_) was ~220 h for Aβ40. D-[D1,A2], D-[E3, F4], D-[R5,H6], D-[H14,Q15], D-[V18,F19], D-[E22,D23], and D-[S26,N27] displayed substantially shorter (<1/2× ) half times (Fig. 3, Fig. S1, and Table 1). D-[V12,H13], D-[N27,K28], and D-[A30,I31], D-[L34,M35], and D-[M35,V36] displayed longer half times, but none were >2× that of wild type. Lag times also were calculated and were found to correlate strongly with t_1/2_ (Fig. S2A). We also calculated the change in fluorescence per unit time (dFU/dt) during the quasi-linear phases of growth for each peptide (Table 1). Interestingly, the same eight peptides that produced the highest FU_max_ values also displayed the highest rates of fluorescence increase, 7–32× that of wild type. The two peptides displaying the lowest FU_max_ values, D-[E22,D23] and D-[S26,N27] also showed the lowest values of dFU/dt. Cross-correlation of the dFU/dt, t_1/2_, and FU_max_ determined by linear fitting to each of the respective scatter plots revealed a correlation (R = 0.73 [0.96 with two outliers removed]) between dFU/dt and FU_max_ (Fig. S3). No correlations were observed dFU/dt and t_1/2_ or between FU_max_ and t_1/2_ FU_max_ (Fig. S3).

**Table 1:** Monitoring fibril formation by thioflavin T (ThT) fluorescence.

Peptide	Metric^1^	Positions of D-amino acids																				
		WT	1,2	3,4	5,6	7,8	10,11	12,13	14,15	16,17	18,19	20,21	22,23	23,24	26,27	27,28	30,31	31,32	34,35	35,36	39,40	41,42
**Aβ40**	**FU**_**max**_	196	320	335	180	317	236	1434	130	2900	1773	1657	1616	76	15.7	1344	143	215	1145	348	597	N/A
	**t**_**1/2**_	219	82	96	70	198	229	290	141	228	148	206	131	226	115	269	262	187	246	296	213	N/A
	**dFU/dt**	0.86	2.20	1.70	1.21	0.90	0.98	6.97	0.85	10.72	10.46	8.97	27.24	0.34	0.07	9.48	0.48	1.40	5.44	2.96	6.17	N/A
		**WT**	**D3**	**D4**	**D16**	**D17**	**D26**	**D27**	**D28**													
	**FU**_**max**_	125	225	192	38	1909	29	28	276													
	**t**_**1/2**_	160	163	117	128	136	115	121	112													
	**dFU/dt**	0.66	1.16	0.95	0.13	11.25	0.12	0.10	2.39													
**Aβ42**		**WT**	**1,2**	**3,4**	**5,6**	**7,8**	**10,11**	**12,13**	**14,15**	**16,17**	**18,19**	**20,21**	**22,23**	**23,24**	**26,27**	**27,28**	**30,31**	**31,32**	**34,35**	**35,36**	**39,40**	**41,42**
	**FU**_**max**_	132	87	86	88	108	117	48	354	74	113	235	82	127	192	58	108	60	137	364	151	226
	**t**_**1/2**_	90	103	82	106	88	201	153	229	104	122	190	68	108	247	128	215	133	133	154	162	123
	**dFU/dt**	0.71	0.44	0.62	0.41	0.67	0.96	0.15	2.64	0.47	0.75	0.61	0.36	0.50	1.35	0.20	0.40	0.19	0.61	2.35	1.02	0.94
		**WT**	**D12**	**D13**	**D14**	**D15**	**D20**	**D21**	**D26**	**D28**	**D35**	**D36**										
	**FU**_**max**_	77	76	59	352	58	605	29	123	103	317	123										
	**t**_**1/2**_	45	214	136	172	82	78	107	173	147	133	101										
	**dFU/dt**	0.50	0.32	0.32	0.98	0.33	3.23	0.09	0.31	0.55	1.24	0.78										

Wild type, doubly and singly substituted Aβ40 and Aβ42 peptides at concentrations of 40 or 20 μM, respectively, were co-incubated with 120 μM ThT in 10 mM sodium phosphate, pH 7.4, at 37°C with shaking.^1^Continuous monitoring of fluorescence was done until plateau fluorescence levels had been reached, after which maximum fluorescence (FU_max_; FU), the time at which 1/2 FU_max_ was observed (t_1/2_; h), and the rate of increase in fluorescence (dFU/dt; FU·h^−1^) were determined. N/A is “not applicable.”

In experiments using di-D-amino acid substituted Aβ42 variants, the FU_max_ range was ~50–350, approximately an order of magnitude lower than that of the Aβ40 peptides. We note that the Aβ42 concentration used in these experiments (20 µM) was half that of the concentration used in the Aβ40 experiments (40 µM). However, this difference alone cannot account for the magnitude of difference in FU_max_^39^. Aβ42 displayed a maximum fluorescence intensity of ≈130 FU, thus the relative differences in intensity between the substituted peptides and wild type Aβ42 often were much smaller than in the Aβ40 case. D-[M35,V36] and D-[H14,Q15] produced the highest intensities, ≈3 × that of wild type Aβ42. The intensities of D-[I41,A42], D-[F20,A21], and D-[Ser26, Asn27] were 1.5 × that of wild type Aβ42. D-[V12,H13], D-[Asn27,Lys28], and D-[Ala31, Ile32] had the lowest maximal intensities. The remaining peptides fluoresced with intensities less than or equal to that of wild type Aβ42. The t_1/2_ for WT Aβ42 was 90 h (see Fig. S2B for associated lag times). With the exception of D-[Phe20, Ala21], which had a t_1/2_ of 190 h, all of the other substituted peptides displayed t_1/2_ values of ~100 h. D-[Y10,E11], D-[H14,Q15], D-[S26,N27] and D-[A30,I31] assembled the slowest, with half times that all were >200 h. Only D-[E22,D23] had a half time that was substantially lower than that of WT Aβ42 (68 vs. 90 h). The highest dFU/dt value observed, that of D-[H14,Q15], was 2.64, ~10-fold lower than the highest values observed with the di-substituted Aβ40 peptides. Most di-D-amino acid substitutions produced lower dFU/dt values and increased half times. As with the doubly substituted Aβ40 peptides, when we cross-correlated FU_max_, t_1/2_, and dFU/dt, the only significant correlation observed was between FU_max_ and dFU/dt (R = 0.91) (Fig. S3).

### Oligomerization of mono-D-amino acid substituted Aβ

**Figure 4:**
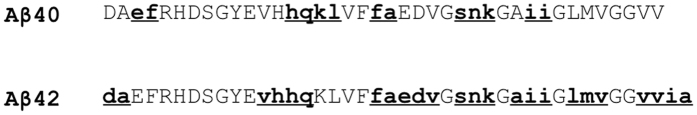
Locations of single D-amino acid substitutions in Aβ40 and Aβ42. Each position in which a single D-amino acid substitution was made is indicated by bolded, underlined, lower-case letters. Thirteen Aβ40 peptides and 24 Aβ42 peptides were studied.

We next synthesized a series of mono-D-substituted peptides (13 for Aβ40, 24 for Aβ42) (Fig. 4). These peptides were chosen based on the results of the PICUP and ThT experiments, which revealed the dipeptide regions of Aβ that most affected oligomerization and β-sheet formation. For each of these dipeptide regions, we synthesized the two corresponding mono-substituted peptides. We then studied oligomerization behaviors using PICUP, SDS-PAGE, and silver staining. We included non-cross-linked peptides in each case because prior studies had shown that Aβ42, but not Aβ40, formed SDS-induced trimers (predominately) and tetramers^9^. We wanted to determine if and how D-amino acid substitution might affect this process. Notwithstanding the prior reported lack of SDS effects on Aβ40 oligomer formation during SDS-PAGE, we included the non-cross-linked Aβ40 peptides as a control and to determine if the prior results were reproducible.

**Figure 5:**
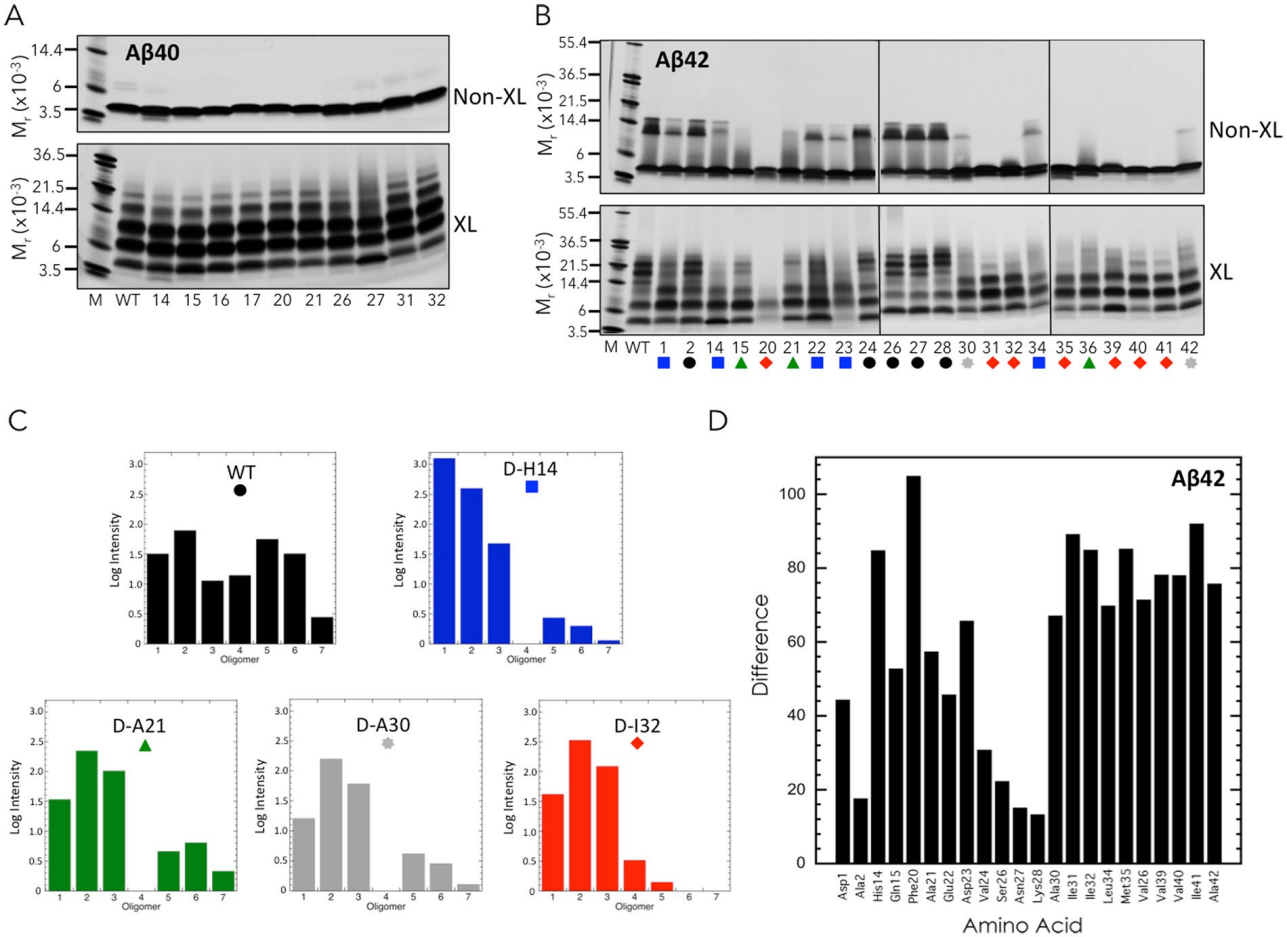
Oligomerization of single D-amino acid substituted Aβ. Non-cross-linked and cross-linked Aβ40 (**A**) and Aβ42 (**B**) were analyzed using SDS-PAGE and silver staining (colored symbols represent different classes of oligomerization; see text). Gels are representative of results in each of three independent experiments. (**C**) Oligomer frequency distributions. Each histogram and its color correspond to one of the five classes of oligomerization pattern shown in panel (B). (**D**) Histogram of the difference metric for Aβ42 variants (see Materials and Methods). Amino acid position is indicated on the abscissa. The sum of the absolute values of the differences in intensities of wild type bands compared with bands in substituted Aβ42 peptides is indicated on the ordinate.

Non-cross-linked Aβ40 displayed an intense monomer band, along with a faint doublet band migrating at an *M*_*r*_ consistent with that of dimer (Fig. 5A). This faint doublet also was observed in the lanes of the D-H14, D-N27, D-I31, and D-I32 peptides, but was not readily visible in other lanes. We also observed a prominent band with the D-H14 peptide that migrated below the monomer band.

Cross-linked Aβ40 produced an oligomer distribution comprising predominately dimers → tetramers, with less intense pentamer and hexamer bands (Fig. 5A, Aβ40, XL). D-N27 showed the greatest difference in oligomer distribution compared to Aβ40, with smeared tetramer, pentamer, and hexamer bands. D-H14 and D-Q15 displayed less intense tetramer and pentamer bands, as well as bands at an M_r_ lower than monomer. This band was particularly distinct in the D-H14 lane, but also was present to varying degrees in other lanes. Relatively small amounts of monomer were observed with D-F20, D-A21, D-S26, D-I31, and D-I32 peptides. Although the nominal amounts of peptide loaded into each well were identical, variations in total band intensity can occur. For this reason, we performed densitometry on each lane and calculated the normalized intensities of bands (see examples in Fig. S4). These intensities were consistent with the observations presented above.

Non-cross-linked Aβ42 produced prominent monomer and trimer bands, and a sharp tetramer band of lower intensity (Fig. 5B non-XL). The trimer band had a characteristic trapezoidal shape^9^. The substituted peptides displayed a variety of patterns, ranging from indistinguishable or similar to Aβ42 (D-A2, D-S26, D-V24, D-N27, and D-K28: black circles) to the presence of only an intense monomer band (D-F20, D-I31, D-I32, D-M35, D-V39, D-V40, and D-I41: red diamonds). Some peptides had relatively little trimer and tetramer (D-D1, D-H14, D-E22, D-D23, and D-L34: blue squares), only small amounts of trimer (D-A30 and D-A42: grey stars), or monomer and a faint band migrating just above (D-Q15, D-A21, and D-V36: green triangles).

Cross-linking of Aβ42 produced a characteristic oligomer distribution comprising predominately dimers → heptamers with a node at pentamer/hexamer^9^. The oligomer distribution of D-F20 differed most from wild type Aβ42 (as it did in its un-cross-linked state), exhibiting only faint monomer, relatively darker dimer, and faint trimer bands. [The low band intensities were not due to material not entering the separating gel, as no staining was observed at the junction of stacking and separating gels.] Interestingly, this variant was the most difficult to solubilize and its oligomer distribution was unique among all the peptides studied. The extent of the effects of D-amino acid substitution in the cross-linked states was very similar to that in the non-cross-linked states (cf. Fig. 5b non-XL vs. XL). If a particular peptide demonstrated essentially complete elimination of oligomers in the non-cross-linked states, this same peptide, when cross-linked, produced distributions in which the predominant oligomer states were dimer and trimer (e.g., see the substitutions from positions 30 → 42), along with faint tetramer, pentamer, and hexamer bands in some samples. Virtually no heptamer bands were visible. Relative to the oligomer distribution produced by WT Aβ42, the D-A1, D-H14, and D-Q15 variants displayed more intense dimer and trimer bands with less intense tetramer, pentamer, and hexamer. Several oligomer distributions were devoid of a tetramer band (D-Q15 and D-A21). Others had very faint monomer bands compared to Aβ42 (D-F20, D-D23, D-M35, D-V36, and D-V40). The D-S26, D-N27, and D-K28 peptides produced distributions that were quite similar to that of WT Aβ42. Densitometric analyses of the normalized band intensities of all peptides were consistent with the observations of relative band intensities described above. Figure 5C presents histograms of representative examples for each of the five classes shown in Fig. 5B.

We created a difference metric (see Materials and Methods) to facilitate quantitative comparisons of the oligomerization behaviors of all the Aβ42 peptides relative to that of wild type Aβ42. A histogram of this metric is presented in Fig. 5D, which illustrates clearly the fact that D-F20 produced the most divergent oligomer distribution. D-H14 also was quite divergent. Two other features of note are the modest differences displayed by D-S26, D-N27, and D-K28 and the strong effects on oligomerization of C-terminal substitutions (D-A30 through D-A42).

### Thioflavin T fluorescence of single D-amino acid substituted Aβ

**Figure 6:**
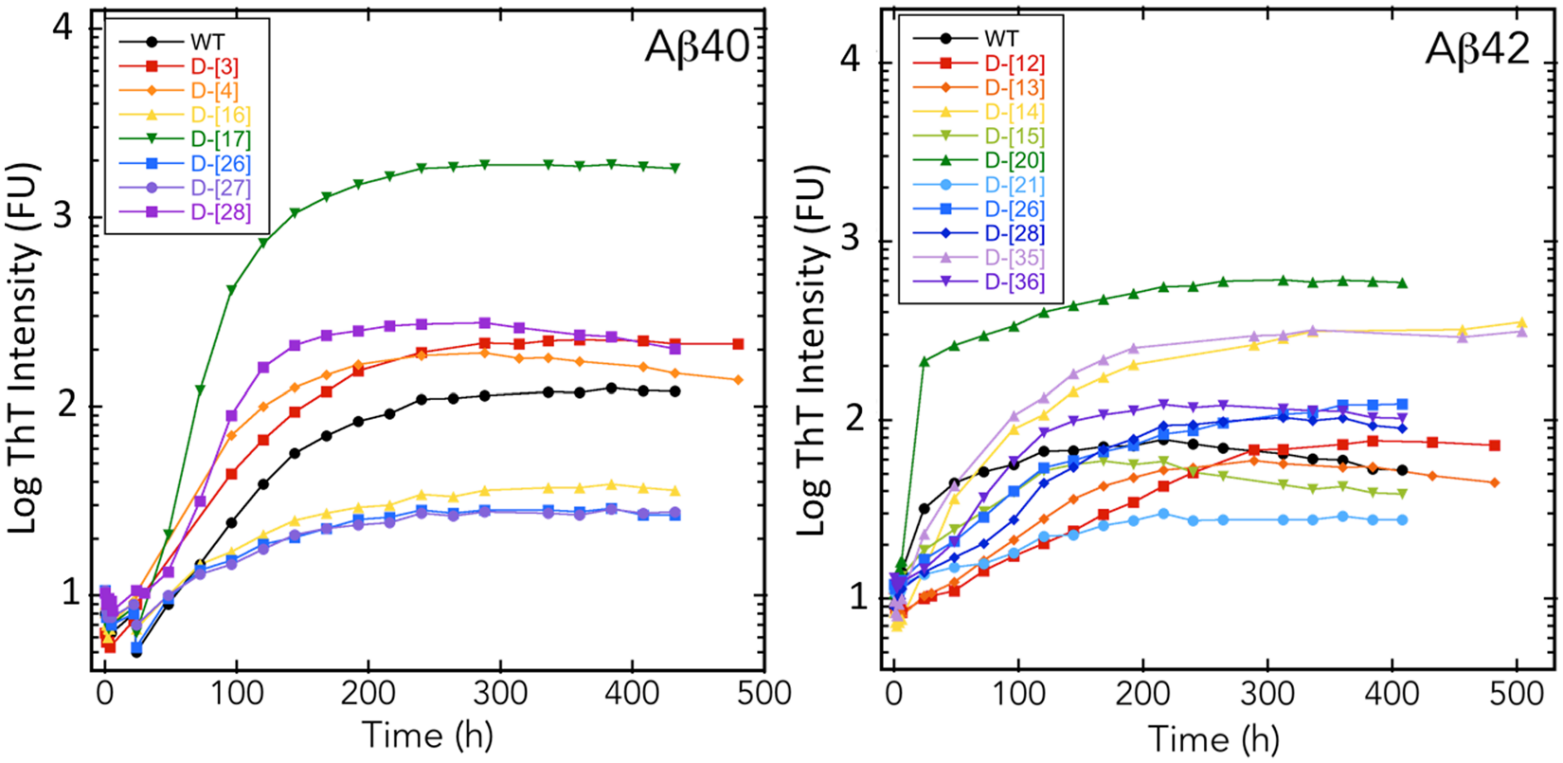
Fibril formation kinetics of single D-amino acid substituted Aβ. Peptides (40 μM Aβ40, 20 μM Aβ42) were mixed with Thioflavin T in 10 mM sodium phosphate, pH 7.4, and incubated with shaking at 37 °C. (**A**) Aβ40 with single D-amino acid substitutions. (**B**) Aβ42 with single D-amino acid substitutions. The peptides examined are shown in the boxes inside each panel. Note that semi-log plots are shown. (Figure S5 shows linear plots).

The time dependence of ThT fluorescence of the singly substituted Aβ40 peptides produced three types of plots relative to that of wild type Aβ (Fig. 6): (1) substantially increased fluorescence; (2) grossly similar; or (3) substantially decreased fluorescence. The biggest contrast in assembly was exhibited by D-L17, which had a final fluorescence intensity of ≈1900, which was 16× higher than that of the wild type peptide, and a t_1/2_ = 136 h, compared to 150 h for wild type (Table 1; Fig. S5). [Note that the data in Fig. 6 are presented on a semi-log plot, which may give the appearance that the t_1/2_ time difference is much smaller than indicated in the text]. D-K16, D-S26, D-N27 displayed substantially lower final ThT fluorescence intensities (38, 29, and 28 FU, respectively) (Fig. 6, Table 1). With the exception of D-E3, which had a half time equivalent to that of WT Aβ40, all the other peptides had shorter half times.

The singly substituted Aβ42 peptides demonstrated higher, lower, and equivalent assembly characteristics relative to the wild type peptide. D-F20 produced a hyperbolic increase (no discernible lag phase) in fluorescence that peaked at 605 units, ~8× those of Aβ42. D-H14 and D-M35 produced very similar curves, each of which reached maximum fluorescence levels of 350 and 320 units, ~4.5× those of Aβ42 (Fig. 6, Table 1, and Fig. S5). D-A21 displayed the largest decrease in final fluorescence intensity, which was ~5× lower than that of wild type. The remaining peptides produced final fluorescence intensities either equal to those of wild type or only modestly (|Δ| < 2) higher or lower.

### Secondary structure of single D-amino acid substituted Aβ

**Figure 7:**
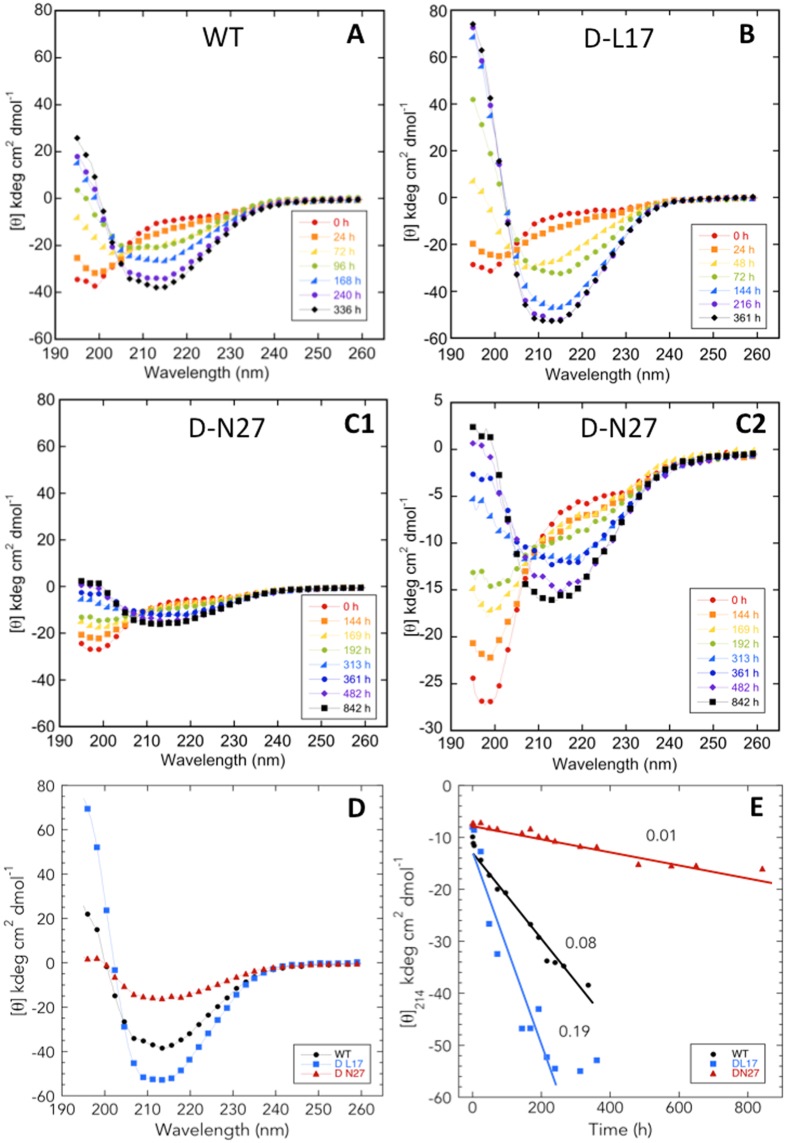
Secondary structure dynamics of WT and singly substituted variants of Aβ40. Forty μM Aβ40 was prepared in 10 mM sodium phosphate, pH 7.4, and incubated with shaking at 37 °C. Secondary structure dynamics were assessed using CD during 842 h of incubation. Panels are (**A**) WT, (**B**) D-L17, and (**C1** and **C2**) D-N27. The data from panel C1 are plotted in panel C2 with a narrower molar ellipticity range to make spectral comparison easier. (**D**) Spectra of WT and substituted variants at the end of secondary structure changes. (**E**) Values of [θ]_214_ are plotted versus incubation time (h). Lines obtained from linear regression analysis of the data were used to determine assembly rates dθ/dt. Numbers adjacent to each line are the calculated rates in kdeg cm^2^ dmol^−1^/hour.

To examine the effects of D-amino acid substitutions on secondary structure dynamics, we monitored the assembly of Aβ40 (D-L17 and D-N27) and Aβ42 (D-H14, D-F20, D-A21, D-M35) peptides using circular dichroism (CD) spectroscopy (Fig. 7A–C). These peptides were selected for study because the substitutions produced the largest effects on oligomerization and emanation of β-sheets. WT Aβ40 and its singly substituted D-L17 and D-N27 forms all initially displayed spectra consistent with primarily statistical coil (SC) structure (monotonic decrease in [θ] from 260 to ~200 nm, at which point an upward inflection occurs). All three peptides then underwent a time-dependent local two-state (isodichroic point at ≈205 nm) transition to β-sheet structure. Although the final structures of each peptide exhibited differences in intensity, their spectral shapes were all similar (Fig. 7D). This transition occurred most rapidly (>2× that of WT) for the D-L17 peptide (Fig. 7E), an observation consistent with this peptide’s especially rapid rate of increase in ThT fluorescence and its very high final fluorescence intensity (Fig. 6). D-N27, the peptide showing the lowest rate (<1/7 that of WT) of ThT fluorescence change and the lowest final fluorescence intensity, also displayed the slowest transition to β-sheet, 1/8 the rate of wild type.

**Figure 8:**
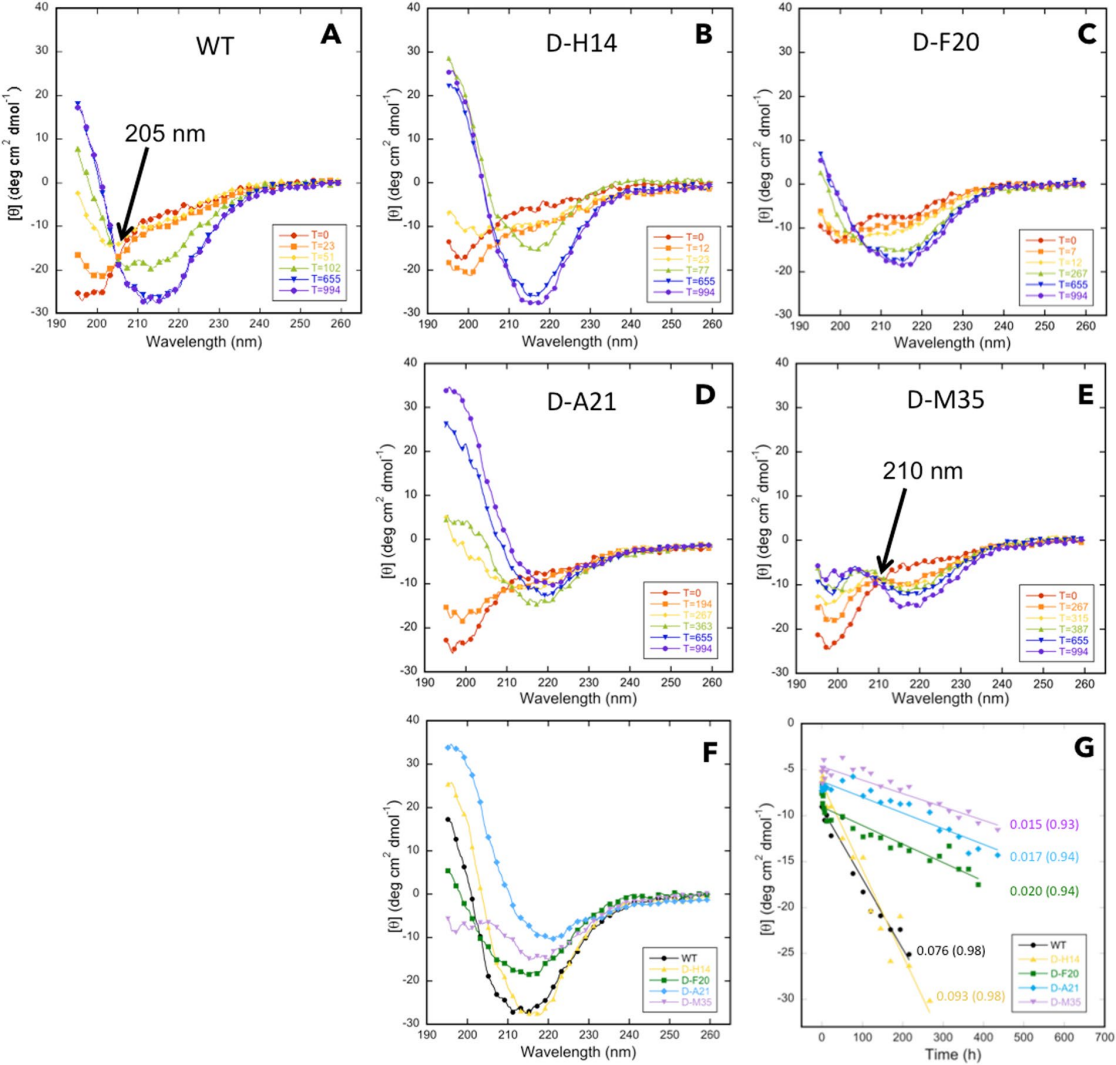
Secondary structure dynamics of WT and singly substituted variants of Aβ42. Twenty μM Aβ42 was prepared in 10 mM sodium phosphate, pH 7.4, and incubated with shaking at 37 °C. Secondary structure dynamics were assessed using CD during 994 h of incubation. Panels are (**A**) WT, (**B**) D-H14, (**C**) D-F20, (**D**) D-A21, and (**E**) D-M35. (**F**) Spectra of WT and substituted variants at the end of the assembly process. (**G**) Molar ellipticity for each peptide was determined at that wavelength at which the molar ellipticity was at a minimum in the final secondary structure formed. Lines obtained from linear regression analysis of the data were used to determine assembly rates dθ/dt. Numbers adjacent to each line are the calculated rates in kdeg cm^2^ dmol^−1^/hour.

Studies of the WT Aβ42 and its singly substituted D-H14, D-F20, D-A21, and D-M35 forms showed initial spectra consistent with statistical coil structure, and subsequent coil → β-sheet transitions (Fig. 8A–E). These transitions have been shown to involve a transitory α-helix-containing intermediate^40^, the presence of which was indicated by spectra in WT, D-H14, D-A21, and D-F20 obtained at 102 h, 77 h, 363 h, and 267 h, respectively. These spectra displayed inflections at ≈208 and ≈219 nm, ≈209 and ≈220 nm, ≈210 and ≈220 nm, and ≈208 and ≈215 nm, respectively. The relatively small magnitudes of the molar ellipticity values for the D-M35 spectra precluded us from confidently assigning α-helix structure to those peptide conformers. In Aβ42 itself, the existence of an isodichroic point at ≈205 nm is consistent with a local two-state transition^41^. We did observe such a point at ≈210 nm in the D-M35 spectra (Fig. 8E). This suggests that the conformational dynamics of D-M35 also involves a two-state transition. In addition, the 5 nm difference in isodichroic point (205 vs. 210 nm) may mean that the two states in D-35 are not identical to those of Aβ42.

Spectra obtained when conformational changes stopped revealed differences among the peptides (Fig. 8F). WT and D-H14 had high β-sheet content, as indicated by minima at ≈215 nm. Asymmetry in the trough observed in D-H14 between 210 and 225 nm may represent some helical content, but variance in these data is too high to argue this confidently. However, this asymmetry is more obvious in the spectra of D-F20 and D-A21, suggesting that the final conformational states of the assemblies formed by these peptides have substantial β-sheet content but may also contain α-helix. D-M35 may have a similar mixed β-sheet and α-helix final conformation. When we plotted the time dependence of the molar ellipticity of each peptide (choosing [θ]_λ_ at that wavelength (λ) where the spectral minimum was observed for each peptide), we observed rapid initial decreases that were followed by monotonically decreasing quasi-linear phases (Fig. 8G). Linear fitting of these phases revealed that WT and D-H14 transitioned from SC → β-sheet the most rapidly and at approximately the same rate. Each of the other three peptides transitioned much more slowly.

### Morphology of single D-amino acid substituted Aβ

**Figure 9:**
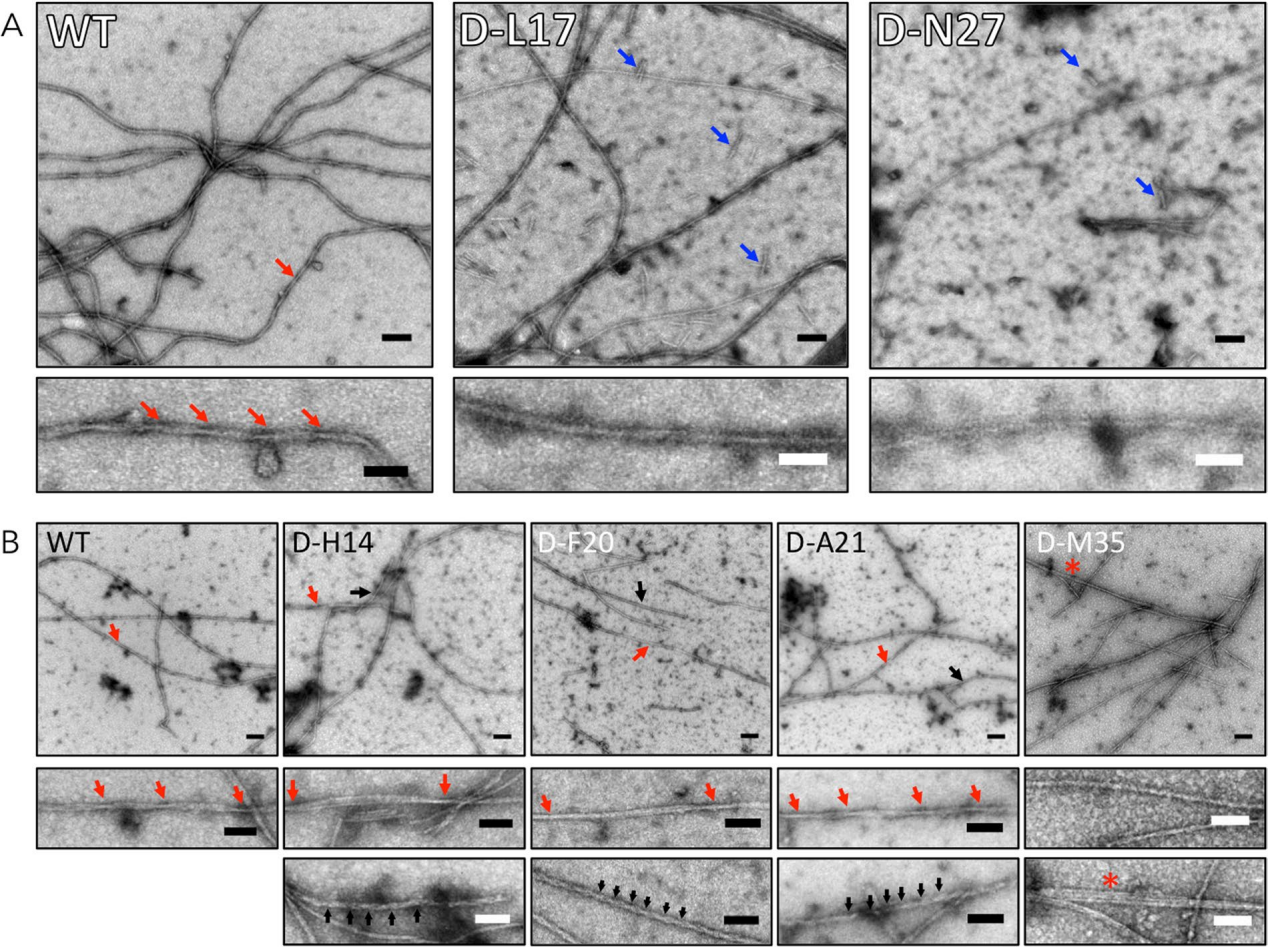
Morphology of WT and singly substituted Aβ40 and Aβ42. Morphology was examined using negative stain transmission electron microscopy. Electron micrographs of fibrils formed by (**A**) WT Aβ40, Aβ40 D-L17, Aβ40 D-N27; and (**B**) WT Aβ42, Aβ42 D-H14, Aβ42 D-F20, Aβ42 D-A21, and Aβ42 M35 are shown. Upper panels show representative regions of the grids. Lower panels are high magnification images of the assemblies indicated by arrows in the upper panels. The colors in the lower panels correspond to those in the upper panels. Scale bars are 100 nm in the upper panels and 50 nm in the lower panels. Arrows in the lower panels indicate the periodicity of helical segments of fibrils. Blue arrows in D-L17 Aβ40 and D-N27 Aβ40 indicate short fibrils. The asterisk in D-M35 Aβ42 indicates a trifilar structure.

To determine the morphologies of the assemblies present after conformational changes ceased (as assessed by CD), we used negative stain transmission electron microscopy (EM) (Fig. 9). Aliquots were removed from the same peptide assembly reactions studied by CD so that direct comparisons were possible. No fibrils were observed in any samples immediately after preparation, with the exception of Aβ42 D-F20 peptide (Fig. S6). Aliquots removed at intermediate and end times all contained fibrils.

End-stage fibrils of WT Aβ40 had average diameters of ~12 nm and displayed a helical twist with a periodicity of 60–140 nm (Fig. 9A, red arrows). When examined at lower magnification, these fibrils were observed to have length exceeding 1 mm (data not shown). Interestingly, D-L17 peptides formed such structures at intermediate times (50 h). D-L17 also displayed monofilar structures with average diameters of ~6 nm as well as shorter filaments ranging in length from 40–100 nm (blue arrows). End-stage D-N27 fibrils had diameters of ~10 nm, slightly narrower than those of WT Aβ40, and lengths exceeding 1 mm. We also observed shorter fibrils that were 40–100 nm in length (blue arrows).

End-stage Aβ42 fibrils were long (>1 mm), had diameters of ≈10 nm, and displayed a helical twist with a periodicity of ~140–250 nm (Fig. 9B, Aβ42, red arrows). We observed two predominant fibril morphologies with D-H14, the first was similar to that of WT Aβ42 (red arrows) whereas the second was characterized by thinner fibrils with average diameters of ~8 nm (black arrows). The thinner filaments were highly twisted with a pitch of ~25 nm. These highly twisted filaments tended to be shorter (average length 450 nm) (Fig. 9B, D-H14). Interestingly, D-F20 and D-A21 fibrils also displayed both types of morphologies as those observed in D-H14, though for D-F20 they appeared earlier. The fibrils observed for D-M35 were relatively straight and also had an average diameter of ≈10 nm. They displayed much less twisting than did fibrils formed by the other peptides. Distinct filaments of ~5 nm in diameter, and with lengths of 40–600 nm, also were observed in the D-M35 sample, as were occasional trifilar structures (Fig. 9B, D-M35, asterisk).

## Discussion

We report here results from experiments designed to reveal the effects on oligomerization, secondary structure dynamics, emanation of β-sheet, and fibril formation of double or single chiral substitutions in Aβ40 and Aβ42. The chiral substitution strategy comprised examination of doubly substituted peptides followed by study of the singly substituted peptides from dipeptide segments found to substantially alter Aβ assembly. A total of 76 different D-amino acid substituted Aβ peptides were studied using four different techniques, photochemical cross-linking (PICUP), ThT binding, CD, EM. The data obtained in our study were voluminous. For this reason, we divide our discussion into two parts. We first review and discuss key results of our experiments. We then conclude by summarizing the most salient findings and discussing their importance with respect to Aβ assembly.

### Oligomerization of substituted peptides

Twenty-one percent (4/19) of the doubly substituted Aβ40 peptides, and sixty-five percent (13/20) of the doubly substituted Aβ42 peptides, produced substantial changes in oligomerization. Changes in Aβ40 included oligomerization inhibition and oligomerization facilitation. Changes in the Aβ42 case were primarily changes in the relative amounts of oligomers of order 2–7 that normally are seen in the oligomerization of Aβ42, with no substantial increases in the amounts of higher order oligomers. We interpret these data to mean that even the subtle structural change produced by chiral substitution of a single amino acid in an Aβ monomer can alter substantially the occupancy frequencies of the different volumes of conformational space accessible to that peptide. This experimental observation is consistent with theoretical considerations of chiral substitution, which propose that such substitutions may change the relative frequencies at which the substituted peptides follow folding trajectories available to their wild type analogues and, in addition, allow them to explore new trajectories^30^. The observation that oligomerization behavior was affected by substitutions in ~3× as many Aβ42 peptides than Aβ40 peptides is consistent with the facts that the topologies of the energy surfaces for Aβ42 and Aβ40 monomer folding are distinct, as are their oligomerization behaviors^9,42^. These data may also reflect the fact that the native Aβ42 monomer possesses greater order than does the Aβ40 monomer^43^. Destabilization of this order by D-amino acid substitution thus would produce larger effects in experiments monitoring peptide structure and dynamics.

Particularly strong effects were seen with substitutions at the C-terminus of Aβ42 (residues 30–42). This region has been shown to contain a turn at Val36-Gly37 that is postulated to stabilize a hydrophobic surface important in intermolecular interactions underlying oligomerization and subsequent fibril formation^43–46^. The results of our studies here with the D-[Val39,V40] and D-[Ile31,Ala42] peptides provide experimental evidence for this postulation. These studies showed that altering the geometry of the apolar side-chains of these amino acids produced oligomer distributions similar to those of Aβ40, not Aβ42. This observation could be due to effects on formation of the postulated hydrophobic surface and its intermolecular interactions. A prior study of oligomerization of singly substituted [Gly41]Aβ42, a much more structurally disruptive substitution, also resulted in Aβ40-like oligomerization^47^. We note that the D-[I31,I32] substitution also inhibited oligomerization of Aβ40, likely because of similar effects on hydrophobic surface stability.

### Emanation of β-sheet structure

We found that chiral substitutions changed the rates of emanation and the final levels of β-sheets. In the case of Aβ40, these changes were quite large and included significant enhancement of β-sheet formation as well as significant inhibition of the process. Some substitutions resulted in changes in these metrics that were as large as 32× or as small as <1/10 that found in the wild type peptides. For all four systems studied, i.e., doubly- and singly-substituted Aβ40 or Aβ42, we found a high correlation between the rate of β-sheet growth (dFU/dt) and final fluorescence intensity (FU_max_) (R = 0.82 for the combined data set (data not shown)), but not between dFU/dt and t_1/2_ or between FU_max_ and t_1/2_. We interpret these results to mean that once fibril nucleation has occurred, the activation energy for monomer addition is relatively low, which likely reflects the facile ability of an incoming monomer and fibril end to interact (high dFU/dt), and in addition, to produce polymeric structures with high affinity for ThT (FU_max_). These metrics do not correlate with t_1/2_, which is determined predominately by lag time (Fig. S2A and B, right panels).

Fibril formation by Aβ40, as monitored using ThT binding, was more sensitive to chiral changes than was oligomerization (as measured by PICUP). Sixty-eight percent (13/19) of Aβ40 substituted peptides showed substantial (>2× or <1/2× relative to wild type) differences in dFU/dt compared with 21% in oligomerization experiments. Substantial differences in dFU/dt were observed in 7/20 (35%) of the substituted Aβ42 peptides. Two D-substituted dipeptide segments were found to produce similar effects in Aβ40 and Aβ42, D23-V24, which inhibited fibril formation, and M35-V36, which substantially increased fibril formation. Inhibition of fibril formation by the D23-V24 peptide may be due to interference with Coulombic interactions between an Asp side-chain in one monomer and a Lys side-chain in a different monomer^48^. Facilitation of fibril formation by the M35-V36 peptide is consistent with the interpretation that chiral changes produce more extensive hydrophobic surfaces that help stabilize both intra- and intermolecular interactions^42,49^. Electron spin resonance studies support the involvement of Met35 in formation of a “C-terminal core” facilitating aggregation^50,51^. The observation that profound inhibition of fibril formation by peptides containing Met35 sulfoxide or sulfone, which make the side chain polar, also supports this interpretation^52,53^.

Correlations were observed between oligomerization and fibril formation. For example, the D-[K16,L17] Aβ40 peptide, which had the highest FU_max_ and dFU/dt values, displayed oligomer distributions with increased amounts of higher order oligomers. The D-[A30,I31] Aβ42 peptide, which had profoundly reduced FU_max_ and dFU/dt values, only produced dimers and trimers in oligomerization experiments. However, such correlations were not observed universally. The D-[E22,D23] Aβ40 had the highest growth rate (32× that of wild type), yet it displayed no significant differences in oligomerization. A similar result was found for Aβ42 D-[H14,Q15], which had the largest dFU/dt among the Aβ42 peptides yet displayed apparent oligomerization inhibition (fewer higher order oligomers). Strong correlation between oligomerization and fibril formation processes supports theories of amyloid peptide assembly suggesting simple, linear pathways^54^. Anti-correlations demonstrate that the subtle changes in primary structure produced by chiral substitution can affect different steps in fibril assembly. They attest to the complexity of the assembly process^23^ and inform us about sites (see below) whose role in oligomerization or higher-order assembly (including, but not restricted to, fibril formation) could be elucidated further using high resolution techniques including NMR, x-ray crystallography, and computer simulation.

Singly substituted peptides were studied to determine how each amino acid within a dipeptide contributed to that dipeptide’s effect on the rate of fibril formation (dFU/dt). Facilitation of fibril formation observed with the Aβ40 dipeptide D-[E3,F4] also was observed with peptides in which only one of the pair was included, showing that each peptide contributed to the effect. The inhibition of fibril formation displayed by the D-[S26,N27] substituted peptide also were recapitulated by the two singly substituted peptides, D-S26 and D-N27. For the D-[N27,K28] dipeptide, which exhibited a strong enhancement of fibril formation (>10× that of wild type), the singly substituted D-K28 peptide showed substantial fibril formation enhancement (~4× that of wild type), consistent with Lys28 being especially important in this process and having the dominant effect in the context of the dipeptide^55^. The results from D-[S26,N27] and D-[N27,K28] are consistent with previous *in hydro* and *in silico* studies which postulated the importance of S26 in V24-K28 turn formation, a structure proposed to be important for the intramolecular nucleation of unfolded Aβ monomer to folded monomer^46,56^. For the D-[K16,L17] peptide, which strongly facilitated fibril formation (>10× that of wild type), the single D-L17 substitution had an even greater effect than the double substitution (≈17× that of wild type). Surprisingly, its D-K16 partner *decreased* the rate by a factor of five, which demonstrates that the effect of one amino acid can dominate over that of its partner, as in the case of D-[N27,K28]. The data also show how sensitive the fibril formation process is to structural changes within the D-[K16,L17] dipeptide segment. Both of these cases apply to fibril formation facilitation. We did not observe a dominant effect for fibril formation inhibition among the peptides studied, but we cannot rule out that this phenomenon might be observed if all singly substituted peptides were studied. Results from studies of secondary structure dynamics (CD) and fibril morphology (EM) of the D-L17 peptide were consistent with the results above. This peptide displayed a rapid SC → β-sheet transition coincident with fibril formation that was much more rapid than that of the wild type peptide, in addition to producing fibrils characterized by relatively narrow fibril diameters (~6 nm).

In the Aβ42 system, the D-[V12,H13] peptide inhibited fibril formation substantially (~5×). Each of its component singly substituted peptides also inhibited, but only by a factor of ≈1/3, suggesting a synergistic effect in the context of the dipeptide segment. [Oligomerization studies were not performed on the singly substituted peptides because their dFU/dt values were identical and were only 2/3 that of wild type. Oligomerization behavior only was studied in those singly substituted peptides showing substantial differences with wild type]. The D-[H14,Q15] peptide facilitated fibril formation, and as with D-[K16,L17] Aβ40, contained amino acids that had opposite effects in the singly substituted state. D-H14 facilitated and D-Q15 inhibited fibril formation, with the facilitation activity predominating in the dipeptide state. We also examined singly substituted versions of the D-[F20,A21] dipeptide, which had little effect on dFU/dt but increased FU_max_ and t_1/2_. The D-F20 peptide produced the highest FU_max_ and dFU/dt values of any of the Aβ42 peptides, either in their doubly or singly substituted forms. However, its transition rate from SC → β-sheet was relatively slow and its final assemblies contained substantial amounts of α-helix structure, which may explain the slow transition. Prior studies have shown that the initial SC → α-helix transition found during Aβ assembly^40^ has a significant effect on assembly rate, depending on the stability of the α-helix state^57^. It seems that the D-substituted peptides that formed ThT positive structures the earliest had a greater likelihood of forming highly twisted fibrils. Fibrils were observed immediately after incubation of Aβ42 D-F20, which is consistent with results from CD analysis, showing that D-F20 already possessed β-sheet content at 0 h and from oligomerization studies showing only low quantities of oligomers forming.

The D-A21 peptide, in contrast, produced the *lowest* FU_max_ and dFU/dt values. This peptide also displayed a substantially reduced SC → β-sheet transition rate. However, in the D-[F20,A21] dipeptide, although dFU/dt was similar to that of wild type, FU_max_ was ~2× higher. Thus, once again, the facilitating effect of a chiral substitution predominated over an inhibitory effect. When we examined the D-K28 peptide component of the D-[N27,K28] dipeptide that caused profound decreases in FU_max_ and dFU/dt, we observed that the activity of this peptide trended toward fibril formation facilitation, but very modestly. This result was consistent with those from study of the equivalent D-[N27,K28]Aβ40 peptide. We found that both D-M35 and D-V36 contributed to the fibril formation facilitation observed with the D-[M35,V36] dipeptide. D-M35 displayed 4× the FU_max_ of wild type and demonstrated a dFU/dt second in magnitude only to that of D-F20. A relatively slow SC → β-sheet transition was exhibited by D-M35 in CD experiments, along with evidence suggesting greater α-helical content in its mature assemblies. As with D-F20, an intermediary SC → α-helix transition, and the presence of α-helix in the mature fibrils, may explain why D-M35 facilitation of fibril formation is coupled with a relatively slow SC → β-sheet transition. A slow initial SC → β-sheet transition and high dFU/dt and FU_max_ are expected if an initial rapid monomer self-association produces structures that must rearrange to assembly efficiently.

## Conclusions

Determination of amyloid β-protein structure-assembly relationships is essential for knowledge-based design of therapeutic agents. We show here that a scanning chiral replacement strategy^30^ enables achievement of this goal. This strategy posits that the subtle changes in amino acid side chain structure produced by D-amino acid substitutions enable determination of sites most crucial in mediating peptide conformational dynamics and assembly. This is because the changes affect only that volume of Ramachandran space occupied by the peptide and not amino acid side chain size, flexibility, hydropathy, charge, or polarizability, changes that are much more structurally disruptive.

**Figure 10:**
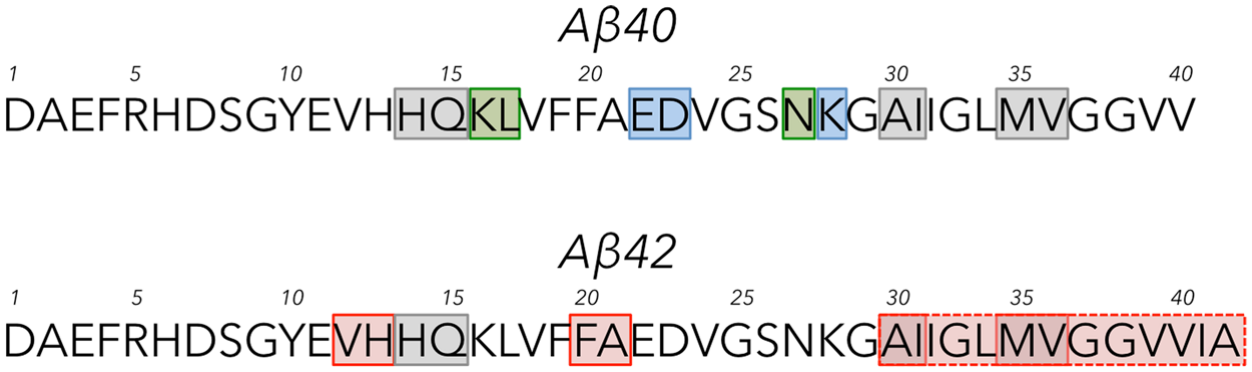
Map of individual amino acids important in controlling Aβ folding and assembly. Amino acids with particularly strong effects on assembly are indicated by shading. Amino acids affecting both Aβ40 and Aβ42 are in grey boxes. Amino acids unique to Aβ40 are in green boxes and those unique to Aβ42 are in red boxes. Amino acids that displayed strong effects by one metric but not all are in blue boxes. The C-terminal region of Aβ42, which we found to be especially sensitive to substitution, is indicated by a red box formed with dashed lines.

Our results (Fig. 10) show that this strategy does indeed provide site-specific information revealing that:

1. A greater number of sites affect Aβ42 assembly than Aβ40 assembly, an observation we interpret as being reflective of the greater structural stability of Aβ42 and thus its higher likelihood of having structural perturbations affect its conformational dynamics and assembly;
2. Substitutions at the same sites in Aβ40 and Aβ42 can produce different effects, emphasizing the fact that the amino acids at these sites may interact with different regions within each respective peptide;
3. Amino acids already demonstrated to be important in controlling Aβ assembly, as evidenced by APP gene mutations that cause singe amino acid substitutions resulting in FAD or CAA (Glu22, Asp23, Lys28), also perturb assembly in their D-enantiomeric forms;
4. Sites shown to be particularly important in facilitating or inhibiting assembly may be appropriate targets for therapeutic agents that inhibit or potentiate, respectively, these effects;
5. Important sites in both Aβ40 and Aβ42 include His14, Gln15, Ala30, Ile31, Met35, and Val36;
6. Sites unique to Aβ40 include Lys16, Leu17, and Asn 27, whereas sites unique to Aβ42 include Phe20 and Ala21.

Recent structural studies on Aβ fibrils offer explanations for the effects of chiral substitutions that we observed. Colvin *et al*. have reported an NMR-derived structure for monomorphic Aβ42 fibrils in which the core building block was a peptide dimer^58^. Intermolecular contacts were observed between Met35 of one monomer and Leu17 and Gln15 of another. Leu17, Phe20, Ala30, Ile31, Val36 formed hydrophobic clusters. Lys28 formed a salt bridge with the C-terminus. In our own experiments, we found that each of these sites was particularly important in conformational and assembly dynamics. Schmidt *et al*. examined Aβ42 fibril structure using cryo-EM and molecular modeling^59^. They described a similar dimer building block. A steric zipper was found to include residues 31–36, three of which we found to substantially affect Aβ dynamics.

More generally, the remarkable sensitivity of the Aβ protein to chiral changes supports the notion that such substitutions in intrinsically disordered amyloid proteins have the ability to alter their assembly pathways and assembly kinetics. Chiral substitutions may substantially inhibit, facilitate, or have no effect on peptide oligomerization, emanation of β-sheet, secondary structure dynamics, or assembly morphology. Comparison of the behaviors of substituted peptides with those of their wild type isoforms allows inferences to be made about the relative ability of the wild type amino acid to mediate folding and assembly.

## Electronic supplementary material

Supplementary Information
