# Supplementary material for: Identification of key regions and residues controlling Aβ folding and assembly
Source: Sci Rep. 2017 Oct 3;7:12434. doi: 10.1038/s41598-017-10845-6 (PMC5626695; doi:10.1038/s41598-017-10845-6)

SUPPLEMENTAL INFORMATON

Identification of key regions and residues controlling Aβ folding and assembly

Eric Y. Hayden[a], Kimberly K. Hoi[a#], Jasmine Lopez[a], Mohammed Inayathullah[c], Margaret M. Condron[a], and David B. Teplow[ab*]

[a]Department of Neurology, David Geffen School of Medicine at UCLA, Los Angeles, CA 90095

[b]Molecular Biology Institute and Brain Research Institute; UCLA, Los Angeles, CA 90095

[c]Biomaterials and Advanced Drug Delivery Laboratory, School of Medicine, Stanford University, Palo Alto, CA 94304, USA

[#]Current address: Department of Pediatrics and Department of Neurology, UCSF, San Francisco, CA 94158

Figure Legends

Figure S1: Fibril formation kinetics of di-D-amino acid substituted Aβ. Peptides (40 μM Aβ40, 20 μM Aβ42) were mixed with Thioflavin T in 10 mM sodium phosphate, pH 7.4, and incubated with shaking at 37° C. The peptides examined are shown in the boxes to the right of each sub-figure. Note that semi-log plots are shown.

Figure S2: Effects of substitutions on lag time in ThT fluorescence studies of fibril formation. Left panels: lag times (ordinate) for (A) Aβ40 and (B) Aβ42 were calculated as discussed in Methods and presented relative to positions of di-D amino acid substitutions (abscissa). Right panels: correlations of lag time and $t_{1/2}$ for (A) Aβ40 and (B) Aβ42. Cross-correlation plots of lag time versus $t_{1/2}$ show that the two metrics are strongly correlated. Correlation coefficients ***R*** were determined by linear fitting to the scatter plots using Kaleidagraph (version 4.5.2) graphing/data analysis software.

Figure S3: Correlations of metrics determined from fibril formation kinetics. Cross-correlation of the dFU/dt, $t_{1/2}$, and FU_{max} determined by linear fitting to each of the respective scatter plots of Aβ40 and Aβ42 revealed a correlation (Aβ40: R=0.73 [0.96 with two outliers (red points) removed] and Aβ42: R=0.91) between dFU/dt and FU_{max}. No correlations were observed between dFU/dt and $t_{1/2}$ or between FU_{max} and t1/2 for Aβ40 or Aβ42.

Figure S4: Oligomerization of single D-amino acid substituted Aβ40. Cross-linked Aβ40 was analyzed using SDS-PAGE and silver staining. Densitometry was performed on representative gels and oligomer frequency distributions were plotted for WT, D-N27 and D-I32.

Figure S5: Fibril formation kinetics of single D-amino acid substituted Aβ. Peptides (40 μM Aβ40, 20 μM Aβ42) were mixed with Thioflavin T in 10 mM sodium phosphate, pH 7.4, and incubated with shaking at 37° C. (A) Aβ40 with single D-amino acid substitutions. (B) Aβ42 with single D-amino acid substitutions. The peptides examined are shown in the boxes inside each panel. This is the linear presentation of the data in Fig. 6.

Figure S6: Morphology of Aβ42 D-F20. Negative stain electron microscopy of Aβ42 D-F20 at t = 0 hrs.

Fig. S1

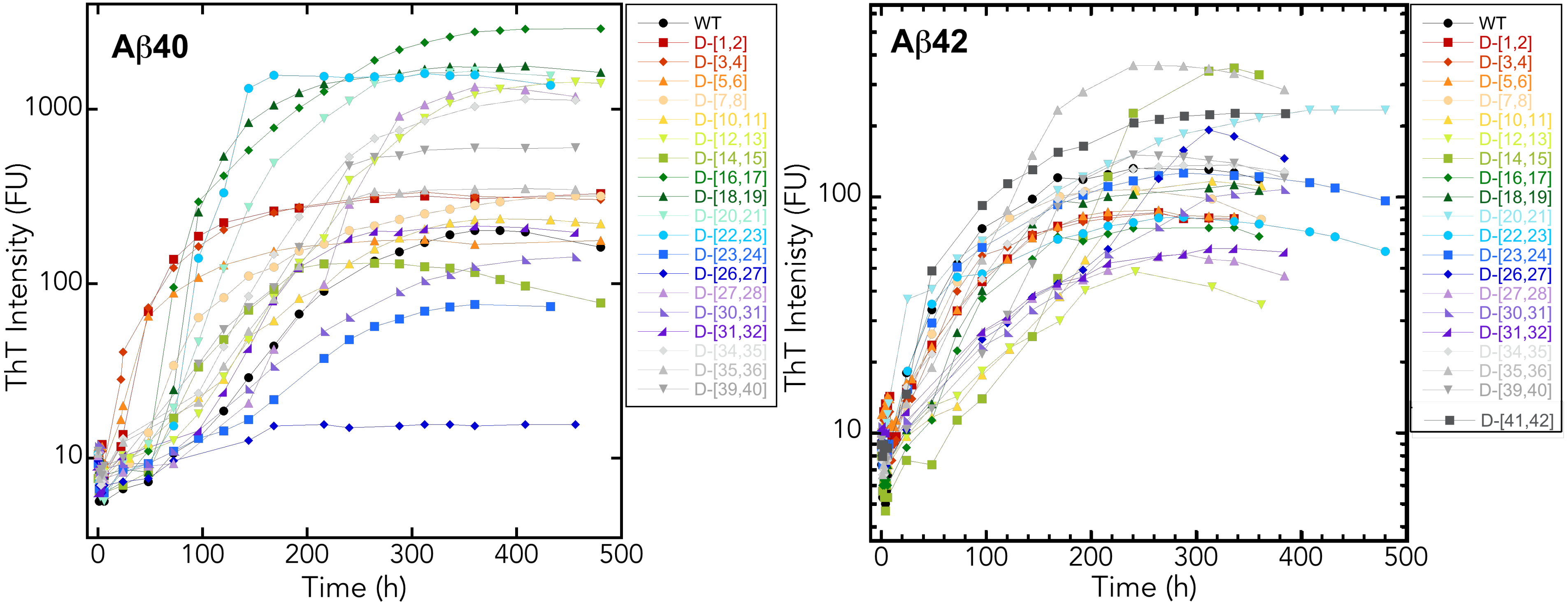
Aβ40
ThT Intensity (FU)
1000
100
10
0
100
200
300
400
500
Time (h)
WT
D-[1,2]
D-[3,4]
D-[5,6]
D-[7,8]
D-[10,11]
D-[12,13]
D-[14,15]
D-[16,17]
D-[18,19]
D-[20,21]
D-[22,23]
D-[23,24]
D-[26,27]
D-[27,28]
D-[30,31]
D-[31,32]
D-[34,35]
D-[35,36]
D-[39,40]
Aβ42
ThT Intenisty (FU)
100
10
0
100
200
300
400
500
Time (h)
WT
D-[1,2]
D-[3,4]
D-[5,6]
D-[7,8]
D-[10,11]
D-[12,13]
D-[14,15]
D-[16,17]
D-[18,19]
D-[20,21]
D-[22,23]
D-[23,24]
D-[26,27]
D-[27,28]
D-[30,31]
D-[31,32]
D-[34,35]
D-[35,36]
D-[39,40]
D-[41,42]

Fig. S2

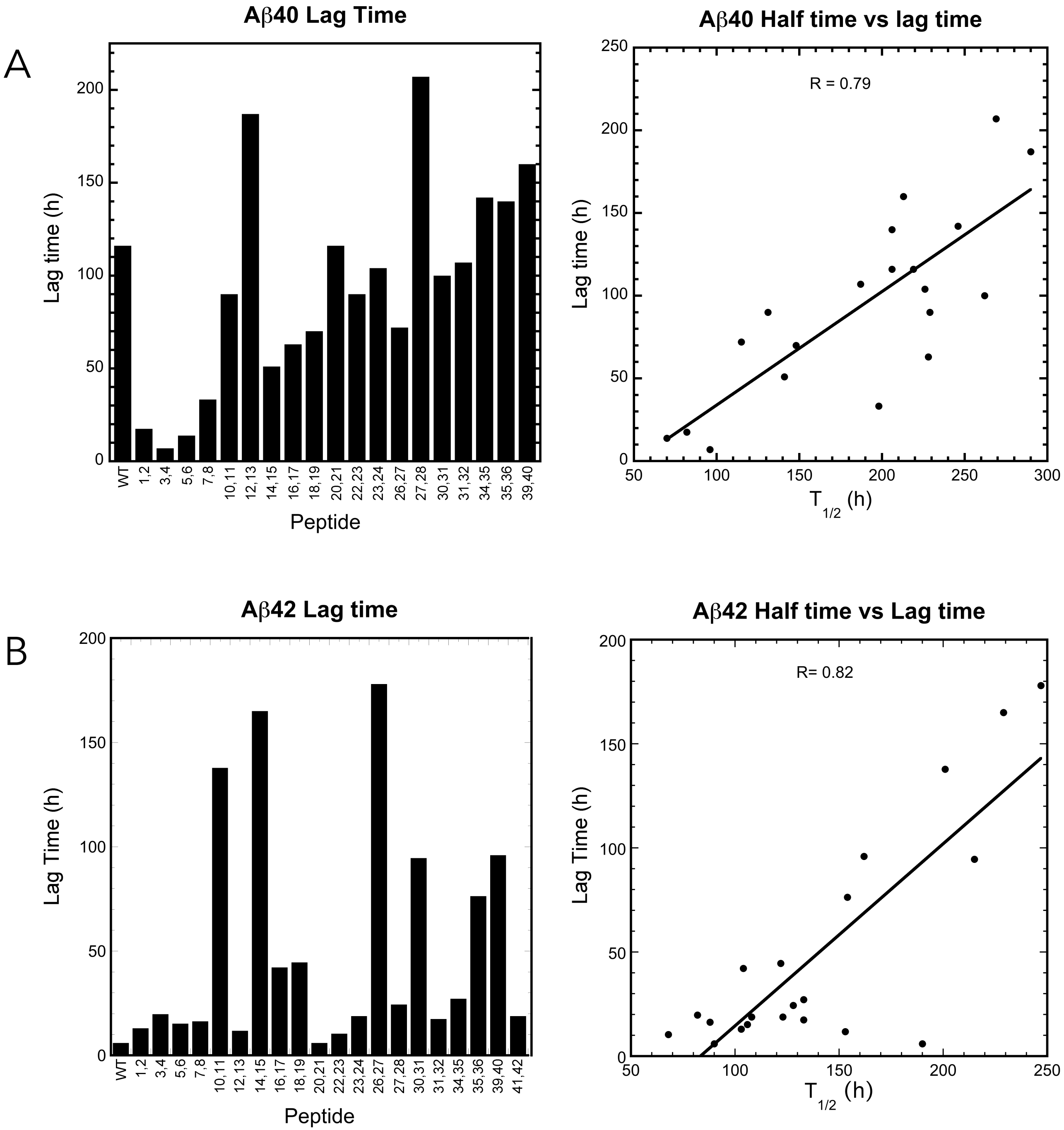
A
Aβ40 Lag Time
Lag time (h)
Peptide
WT
1,2
3,4
5,6
7,8
10,11
12,13
14,15
16,17
18,19
20,21
22,23
23,24
26,27
27,28
30,31
31,32
34,35
35,36
39,40
Aβ40 Half time vs lag time
R = 0.79
Lag time (h)
$T_{1/2}$ (h)
B
Aβ42 Lag time
Lag Time (h)
Peptide
WT
1,2
3,4
5,6
7,8
10,11
12,13
14,15
16,17
18,19
20,21
22,23
23,24
26,27
27,28
30,31
31,32
34,35
35,36
39,40
41,42
Aβ42 Half time vs Lag time
R= 0.82
Lag Time (h)
$T_{1/2}$ (h)

Fig. S3

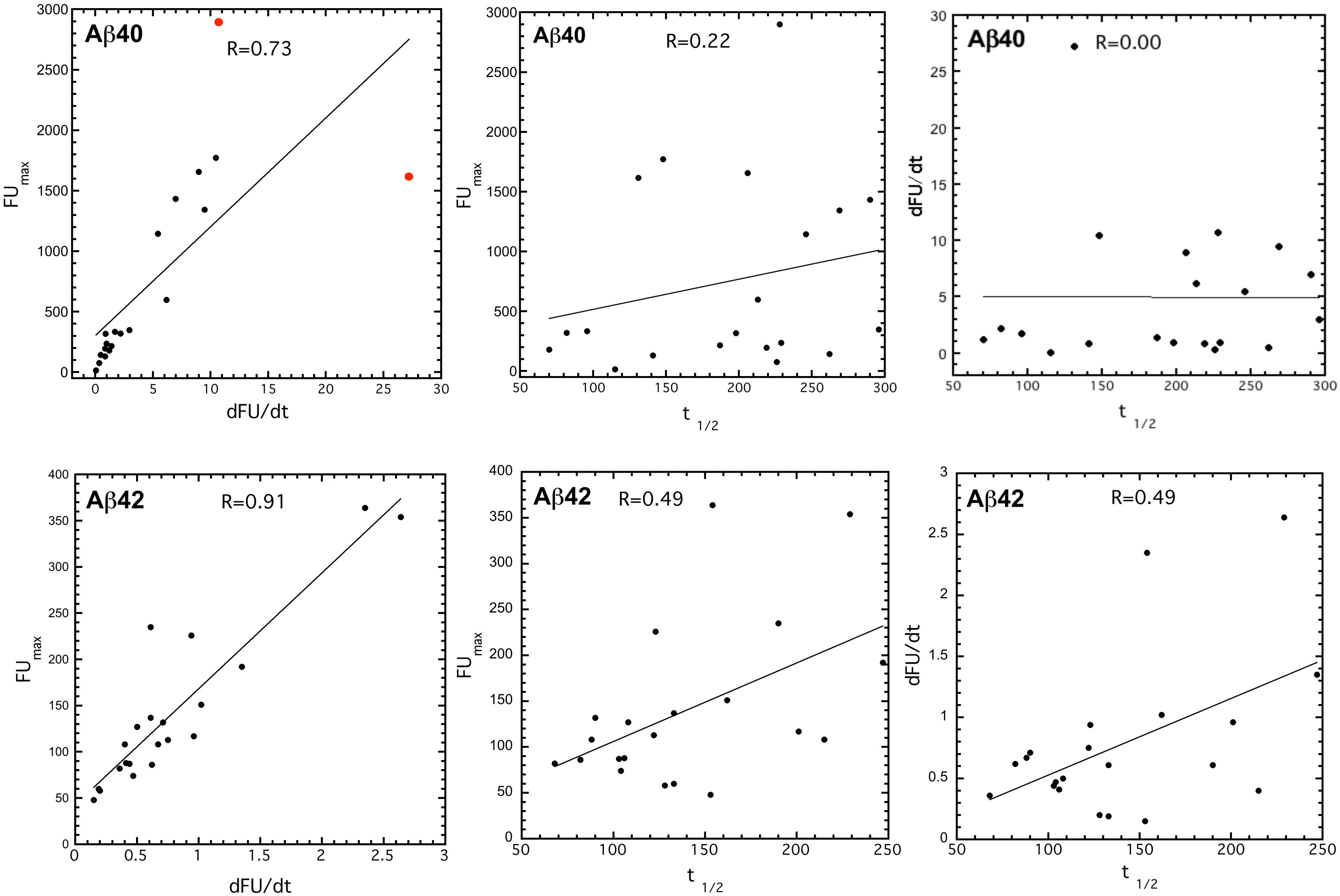

Aβ40
R=0.73
FU max
dFU/dt
Aβ40
R=0.22
FU max
t 1/2
Aβ40
R=0.00
dFU/dt
t 1/2
Aβ42
R=0.91
FU max
dFU/dt
Aβ42
R=0.49
FU max
t 1/2
Aβ42
R=0.49
dFU/dt
t 1/2

Fig. S4

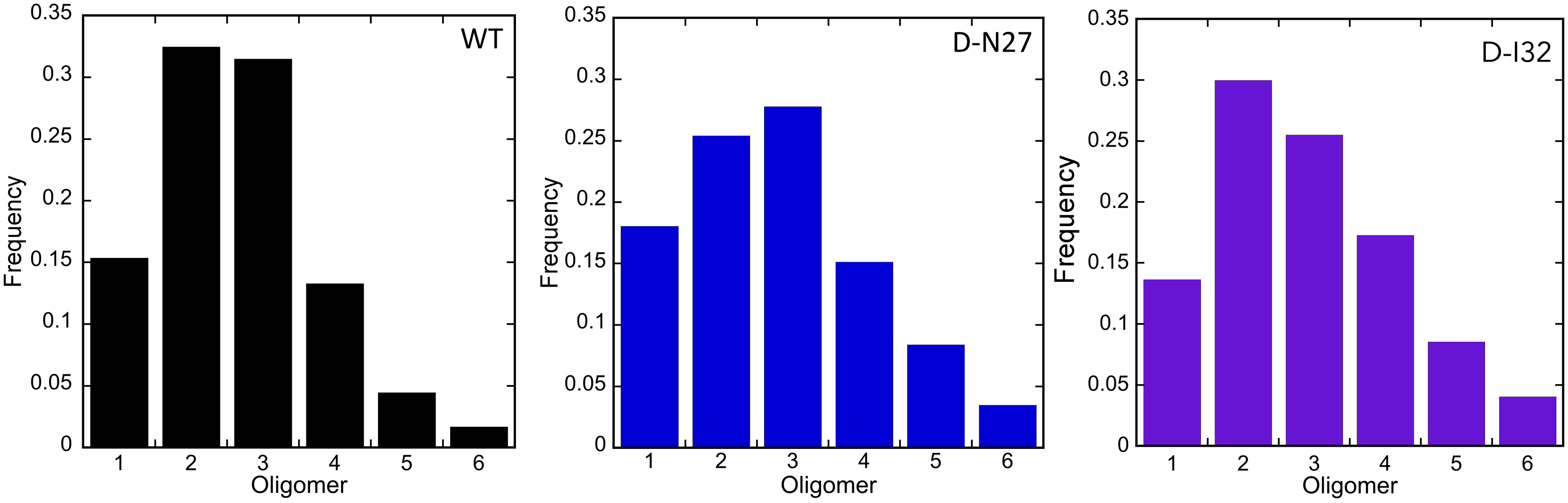
WT
D-N27
D-I32
Frequency
Oligomer
0
0.05
0.1
0.15
0.2
0.25
0.3
0.35
1
2
3
4
5
6

Fig. S5

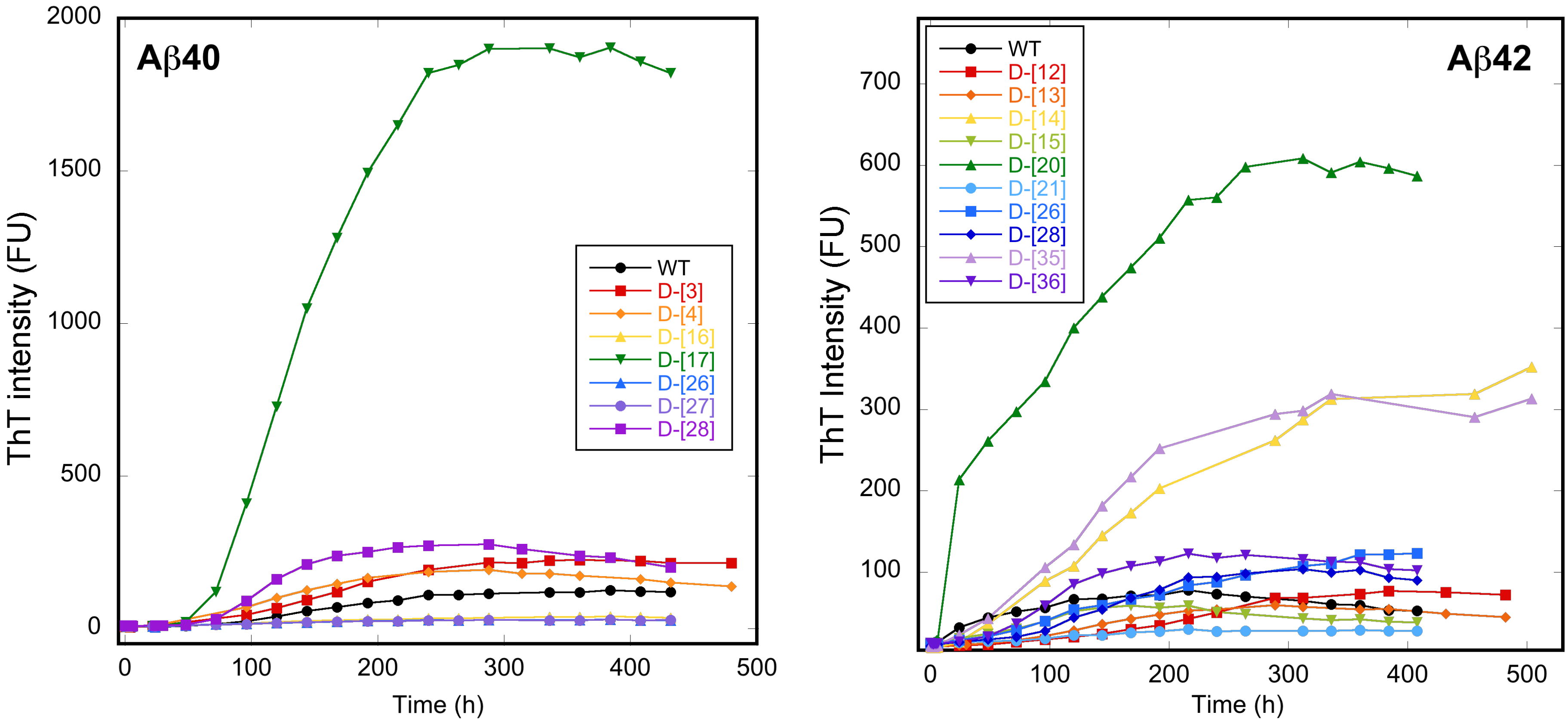

Aβ40
ThT intensity (FU)
Time (h)
WT
D-[3]
D-[4]
D-[16]
D-[17]
D-[26]
D-[27]
D-[28]
0
500
1000
1500
2000
100
200
300
400
Aβ42
ThT Intensity (FU)
WT
D-[12]
D-[13]
D-[14]
D-[15]
D-[20]
D-[21]
D-[26]
D-[28]
D-[35]
D-[36]
600
700

Fig. S6

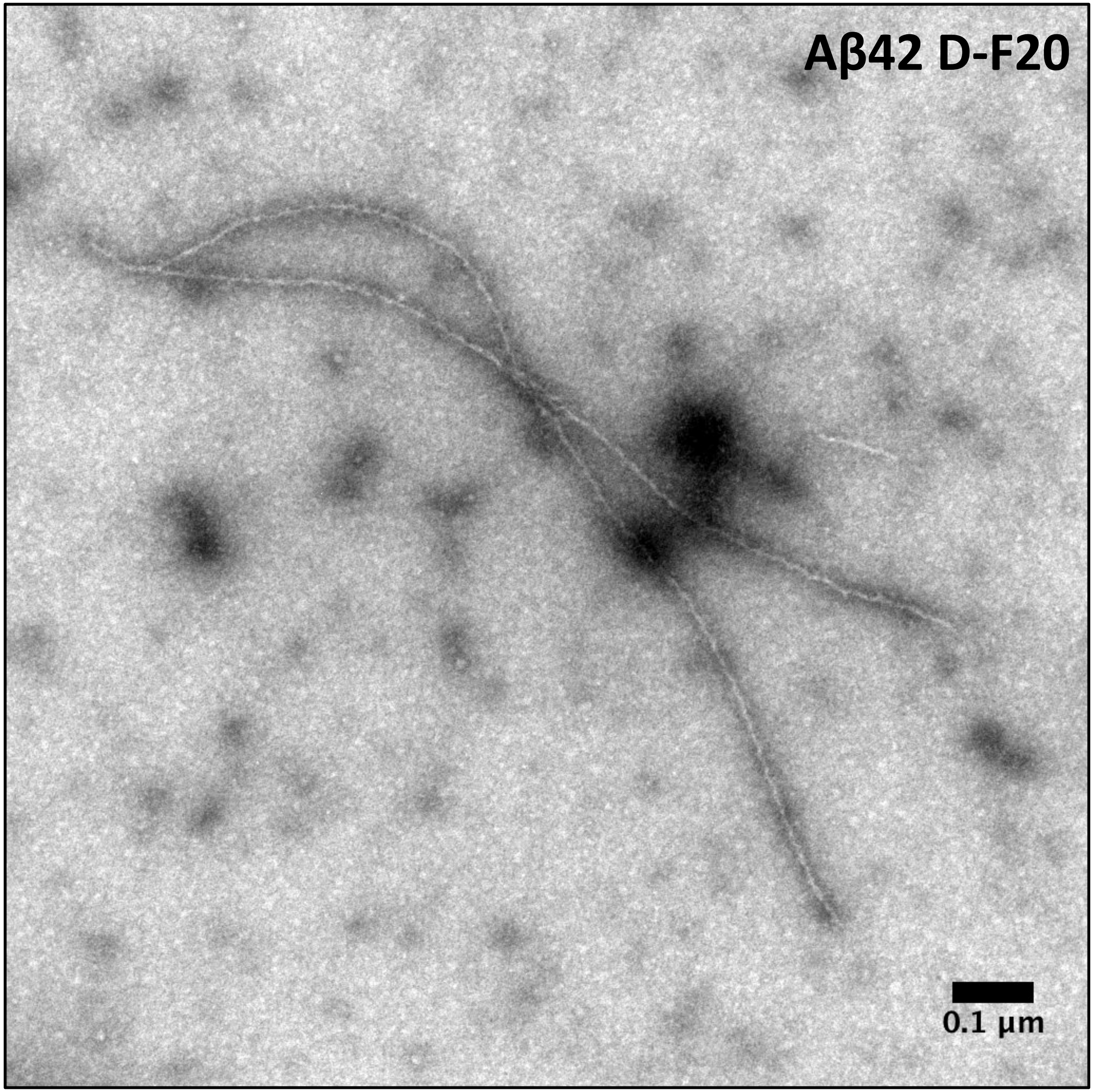

Supplement: Supplementary file 1 — Supplementary Information [file 41598_2017_10845_MOESM1_ESM.pdf]
